# Neutrophils promote T-cell activation through the regulated release of CD44-bound Galectin-9 from the cell surface during HIV infection

**DOI:** 10.1371/journal.pbio.3001387

**Published:** 2021-08-19

**Authors:** Garett Dunsmore, Eliana Perez Rosero, Shima Shahbaz, Deanna M. Santer, Juan Jovel, Paige Lacy, Stan Houston, Shokrollah Elahi

**Affiliations:** 1 Department of Medical Microbiology and Immunology, Faculty of Medicine and Dentistry, University of Alberta, Edmonton, Canada; 2 School of Dentistry, Faculty of Medicine and Dentistry, University of Alberta, Edmonton, Canada; 3 Li Ka Shing Institute of Virology, Faculty of Medicine and Dentistry, University of Alberta, Edmonton, Canada; 4 Department of Medicine, Division of Pulmonary Medicine, Faculty of Medicine and Dentistry, University of Alberta, Edmonton, Canada; 5 Department of Medicine, Division of Infectious Disease, Faculty of Medicine and Dentistry, University of Alberta, Edmonton, Canada; 6 Department of Oncology, Faculty of Medicine and Dentistry, University of Alberta, Edmonton, Canada; Weatherall Institute of Molecular Medicine, UNITED KINGDOM

## Abstract

The interaction of neutrophils with T cells has been the subject of debate and controversies. Previous studies have suggested that neutrophils may suppress or activate T cells. Despite these studies, the interaction between neutrophils and T cells has remained a largely unexplored field. Here, based on our RNA sequencing (RNA-seq) analysis, we found that neutrophils have differential transcriptional and functional profiling depending on the CD4 T-cell count of the HIV-infected individual. In particular, we identified that neutrophils in healthy individuals express surface Galectin-9 (Gal-9), which is down-regulated upon activation, and is consistently down-regulated in HIV-infected individuals. However, down-regulation of Gal-9 was associated with CD4 T-cell count of patients. Unstimulated neutrophils express high levels of surface Gal-9 that is bound to CD44, and, upon stimulation, neutrophils depalmitoylate CD44 and induce its movement out of the lipid raft. This process causes the release of Gal-9 from the surface of neutrophils. In addition, we found that neutrophil-derived exogenous Gal-9 binds to cell surface CD44 on T cells, which promotes LCK activation and subsequently enhances T-cell activation. Furthermore, this process was regulated by glycolysis and can be inhibited by interleukin (IL)-10. Together, our data reveal a novel mechanism of Gal-9 shedding from the surface of neutrophils. This could explain elevated plasma Gal-9 levels in HIV-infected individuals as an underlying mechanism of the well-characterized chronic immune activation in HIV infection. This study provides a novel role for the Gal-9 shedding from neutrophils. We anticipate that our results will spark renewed investigation into the role of neutrophils in T-cell activation in other acute and chronic conditions, as well as improved strategies for modulating Gal-9 shedding.

## Abstract 2

This study shows that HIV-infected individuals have different neutrophil profiles depending on their CD4 T cell count. In particular, neutrophils express high levels of surface Gal-9 but this is shed upon stimulation; this exogenous Gal-9 binds to CD44 on T cells, which promotes LCK activation and subsequently enhances T cell activation.

## Introduction

The activation of neutrophils in circulation causes cellular polarization, which increases the ability of neutrophils to engage in rolling and adhesion to the endothelium of blood vessels, enhancing tissue extravasation [1]. Signaling involved in neutrophil polarization causes the movement of a variety of extracellular adhesion molecules that facilitate neutrophil rolling and adhesion (selectins, integrins, and CD44) [2,3]. Reactive oxygen species (ROS) are involved in efficient chemotaxis and directionality of cytoskeleton remodeling in neutrophils [4].

Neutrophils primarily use glycolysis and the pentose phosphate pathway for energy metabolism [5]. One of the mechanisms of neutrophil protection that relies on glycolysis is ROS production by NADPH oxidation upon stimulation [6]. ROS can also mediate the activation of calcium/calmodulin-dependent protein kinase II (CaMKII), which contributes to cell migration in vascular smooth muscle and apoptosis [7–9]. Upon activation, CaMKII can contribute to neutrophil activation by a variety of mechanisms, for example, CaMKII can activate the inflammatory response proteins AP1, NF-kB, and HDAC4 [10–12]. CaMKII can facilitate CD44 phosphorylation and induces its interaction with the actin cytoskeleton, which is integral for the interaction of CD44 with its ligand hyaluronan [13]. CD44 is expressed on neutrophils and interacts with the extracellular polymer hyaluronan and E-cadherin that are expressed on the surface of endothelial cells and contributes to neutrophil crawling and extravasation [14,15]. In order for CD44 to perform its interaction with hyaluronan, it should interact with the cellular cytoskeleton through a cytoskeleton complex composed of talin-1, RAP-1, and ezrin. Upon binding directly to these proteins, CD44 will move to the trailing end of the cell where it will bind its ligands to promote cell movement [16]. In agreement with these findings, previous studies have shown that during neutrophil polarization, CD44 leaves the lipid rafts and localizes to the uropod during cell polarization [17]. The function of CD44 in neutrophil recruitment is not well understood; however, it is involved in rolling adhesion, implicated in firm adhesion, and is required for neutrophils to enter liver sinusoids [18,19]. CD44 has 3 characterized posttranslational modifications: phosphorylation, palmitoylation, and glycosylation [20]. Phosphorylation of CD44 at the Y395 residue is integral for the movement of CD44 out of the lipid raft and interaction with actin cytoskeleton in neutrophil polarization. The process of CD44 phosphorylation is regulated by CaMKII, calcium release, and oxidation [7,13]. Y395 phosphorylation of CD44 has been speculated to cause a conformational change in the intracellular domain, which could regulate the depalmitoylation (DP) of CD44 [16]. The palmitoylation of CD44 anchors the protein to the lipid raft, similarly to other palmitoylated proteins. Once CD44 is depalmitoylated, it is able to leave lipid rafts and interact with the actin cytoskeleton of the cell. In neutrophils, this process facilitates the engagement of CD44 with its ligand hyaluronan. The role of neutrophils in HIV infection has been contradictory. In general, neutrophil persistence in the site of inflammation can enhance chronic inflammation [21]. The persistent inflammation during suppressive antiretroviral therapy (ART) is a contributing factor to non-AIDS–defining comorbidities [22–24]. For example, neutrophils appear to be more activated in HIV patients with low compared to those with a higher CD4^+^ T-cell count [25]. Moreover, neutrophils from HIV patients with low CD4^+^ T-cell count have increased chemotactic activity [25,26]. Neutrophils may support CD8^+^ T-cell activation in the tissue and contribute to the inactivation of the HIV virion, but their persistence can cause collateral damage [27,28]. In contrast, it is reported that neutrophils via mitochondrial ROS can suppress T-cell functions [29].

T-cell exhaustion is the hallmark of viral infections such as HIV [30]. Exhausted T cells express multiple co-inhibitory receptors (e.g., PD-1, CTLA-4, and TIM-3) [31–34]. However, the expression of co-inhibitory receptors or their ligands on neutrophils in the context of HIV infection has not been well studied. A recent study suggested that neutrophils by the expression of PD-L1 mediate immune suppression in PD-1–expressing T cells [35]. However, this study was focused on low density weight (LDW) neutrophils but not mature neutrophils. The up-regulation of Galectin-9 (Gal-9) on the surface of different immune cells and its elevation in the plasma of HIV-infected individuals have been reported [36,37].

Gal-9 is a beta-galactoside–binding protein in the galectin family of proteins that interacts with different receptors such as CD44, CD137, protein disulfide isomerase (PDI), and TIM-3 [38–40]. Gal-9 can be released by a variety of immune and nonimmune cells, causing a broad spectrum of functions [37]. The plasma Gal-9 increases in HIV-infected patients [41] especially as the CD4^+^ T-cell count decreases [42]. Soluble Gal-9 can activate T cells and is associated with reactivation and transcriptional enhancement of HIV by augmenting extracellular signal–regulated kinase (ERK) signaling through LCK activation [43,44]. In addition to the exogenous Gal-9, surface-bound Gal-9 has been identified on regulatory T cells (Tregs), T cells, natural killer (NK) cells, monocytes, basophils, and mast cells [45–49]. Previous analysis has shown that neutrophils from healthy controls (HCs) are capable of efficiently binding Gal-9; however, the receptor that facilitate this binding activity was not identified [50].

Recent studies have suggested that the interaction of CD44 and Gal-9 on T cells could increase T-cell activation by enhancing ERK signaling [43,44]. In addition, CD44 can stabilize LCK interaction through direct zinc-dependent binding, which is attributed to T-cell spreading and movement [51].

In this study, we show that neutrophils in HIV-infected individuals have differential gene and functional profiling depending on the CD4^+^ T-cell count. Moreover, we describe a novel mechanism and regulatory process that facilitates the release of surface-bound Gal-9 from neutrophils that can activate T cells. Thus, our study presents a novel link between T-cell activation and neutrophils in HIV infection via Gal-9 shedding.

## Results

### Neutrophils from HIV-infected individuals are transcriptionally pro-inflammatory

**Fig 1:**
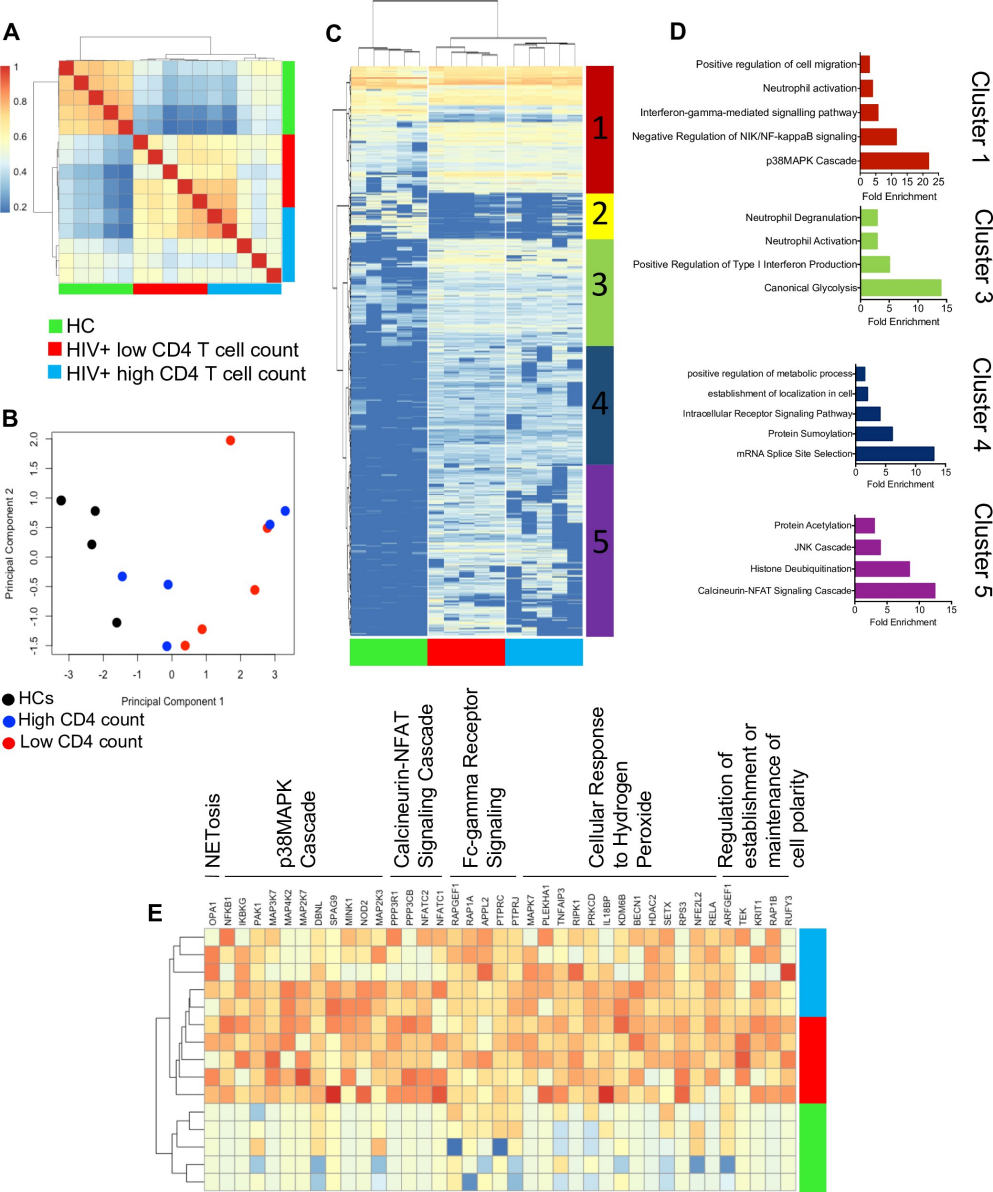
Neutrophils from HIV-infected individuals have a transcriptionally activated phenotype. **(A)** Correlation heatmap representing DEGs in neutrophils from HCs, HIV patients with high and low CD4 T-cell count. **(B)** PCA plot representing the differences between HIV-infected patients with high and low CD4 T-cell count or HCs. **(C)** A clustered heatmap showing the differences between individual genes between HIV patients with low and high CD4 T-cell count and HCs (DEGs have an FDR <0.05 and a log FC >2 or <−2). **(D)** Graphs representing gene ontologies significantly associated with the heatmap clusters (FDR < 0.05). **(E)** Significantly increased genes expressed in neutrophils from HIV patients contributing to gene ontologies. DEG, differentially expressed gene; FC, fold change; FDR, false discovery rate; HC, healthy control; JNK, c-Jun N-terminal kinase; PCA, principal component analysis.

To determine the transcriptional profile of neutrophils in HIV patients, we conducted RNA sequencing (RNA-seq) analysis on total RNA extracted from enriched neutrophils of HIV-infected individuals on ART, with high (>500 cells/μl) and low CD4 T-cell count (<400 cells/μl) in comparison with neutrophils obtained from HCs. When hierarchical clustering was conducted on Euclidean distances between samples, neutrophils from HIV patients clearly showed a different gene expression profile than the HCs (**Fig 1A**). For the most part, healthy samples formed separated branches on a dendrogram, while HIV samples exhibited a more erratic distribution along such dendrogram depending on the CD4 T-cell count (**Fig 1A**). These results were partially recapitulated in principal component analysis (PCA) on the Euclidean distances between samples (**Fig 1B**). In essence, neutrophils from HIV-infected individuals were clearly separated from HCs. However, 3 out of 5 samples from those with a higher CD4 T-cell count were closer to HCs in PCA (**Fig 1B**). To further evaluate the data, significantly differentially expressed genes (DEGs; false discovery rate [FDR] <0.05) with a log fold change difference <−2, or >2, were heatmapped (**Fig 1C**) and compared as shown by volcano plots (**S1A–S1C Fig**). These analyses revealed 5 clusters of DEGs between the groups (HIV patients with high or low CD4 T-cell count and HCs) (**Fig 1C**). To evaluate the biological processes associated with these differentially expressed clusters of genes, the transcripts within each cluster of the heatmap were subjected to gene ontology analysis. Some of the significantly related biological processes were graphically represented demonstrating the processes related to each cluster (**Fig 1D**, **S2A Fig**). Additionally, the genes associated with these ontologies were heatmapped and annotated with their associated gene ontology (**Fig 1E**), which indicated up-regulation of genes associated with NETosis, p38 mitogen-activated protein kinase (MAPK) cascade, calcineurin–nuclear factor of activated T cells (NFAT) signaling cascade, Fc gamma receptor (FcγR) signaling, cellular response to hydrogen peroxide, and the regulation of cell polarity in neutrophils from HIV-infected individuals versus HCs. Furthermore, a wide range of genes associated with neutrophil activation were up-regulated in HIV-infected patients (**S2B Fig**). These observations confirm a more activated transcriptional profile in neutrophils from HIV-infected individuals versus HCs.

### Differential effects of neutrophils on T-cell activation depending on the CD4 T-cell count of HIV-infected individuals

**Fig 2:**
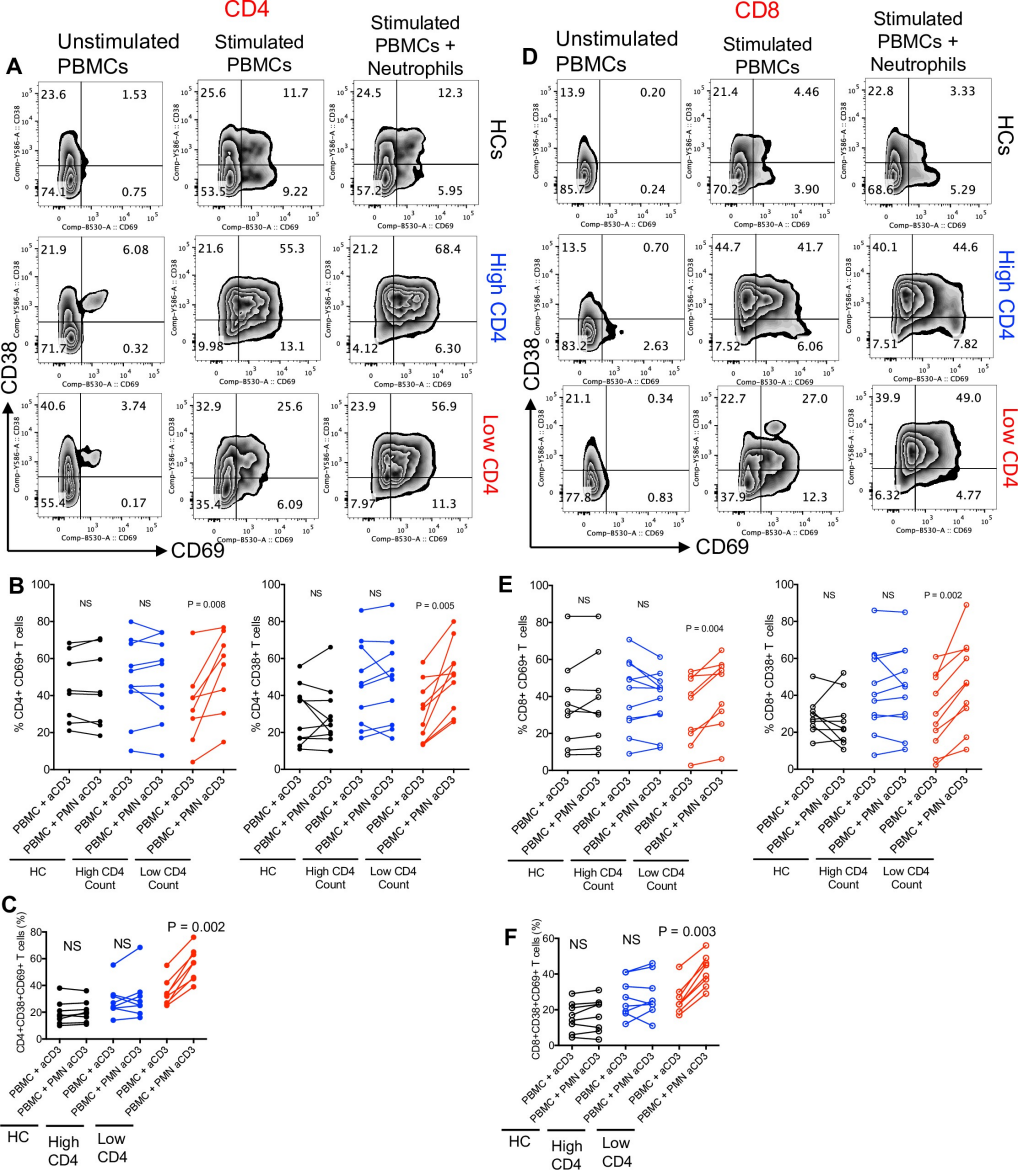
Neutrophils from HIV-infected individuals with low CD4 T-cell count contribute to T-cell activation. **(A)** Representative plots, **(B)** cumulative data showing the percent CD4^+^ T cells expressing CD69 and CD38, and **(C)** co-expression CD69CD38 in the presence or absence of neutrophils (PMN) from HCs, HIV patients with high and low CD4 T-cell count. **(D)** Representative plots, **(E)** cumulative data showing the percent of CD8^+^ T cells expressing CD69 and CD38, and **(F)** co-expression CD69CD38 in the presence or absence of neutrophils from HCs, HIV patients with high and low CD4 T-cell count. The underlying data can be found in S1 Data. HC, healthy control; PBMC, peripheral blood mononuclear cell; PMN, polymorphonuclear.

To evaluate whether neutrophils from HIV patients impact T-cell activation, we activated T cells with anti-CD3/CD28 antibodies using the whole peripheral blood mononuclear cell (PBMC) for 48 hours in the presence or absence of neutrophils from the same individual (1:1 ratio) (**S2C Fig**). We observed a differential effect on the expression of T-cell activation markers (CD69 and CD38) by neutrophils from HIV patients with low versus high CD4 T-cell counts (**Fig 2A–2F**). While neutrophils from HIV-infected individuals with high CD4 T-cells count and HCs did not impact T-cell activation, neutrophils from HIV-infected individuals with low CD4 T-cell count significantly increased the expression of activation markers CD38, CD69, or their co-expression on both CD4^+^ and CD8^+^ T cells in vitro (**Fig 2A–2F**). Similar observations were made for HLA-DR and CD25 co-expression on both CD4^+^ and CD8^+^ T cells (**S3A–S3D Fig**). We also evaluated the co-expression of HLA-DR and CD38 and found the same pattern for both CD4^+^ and CD8^+^ T cells when cocultured with neutrophils (**S3E–S3H Fig**). Although based on the transcriptional profile, neutrophils from HIV-infected individuals were transcriptionally inflammatory; they exhibited differential effects on T-cell activation when cocultured in vitro.

### Neutrophils express differential levels of Gal-9

**Fig 3:**
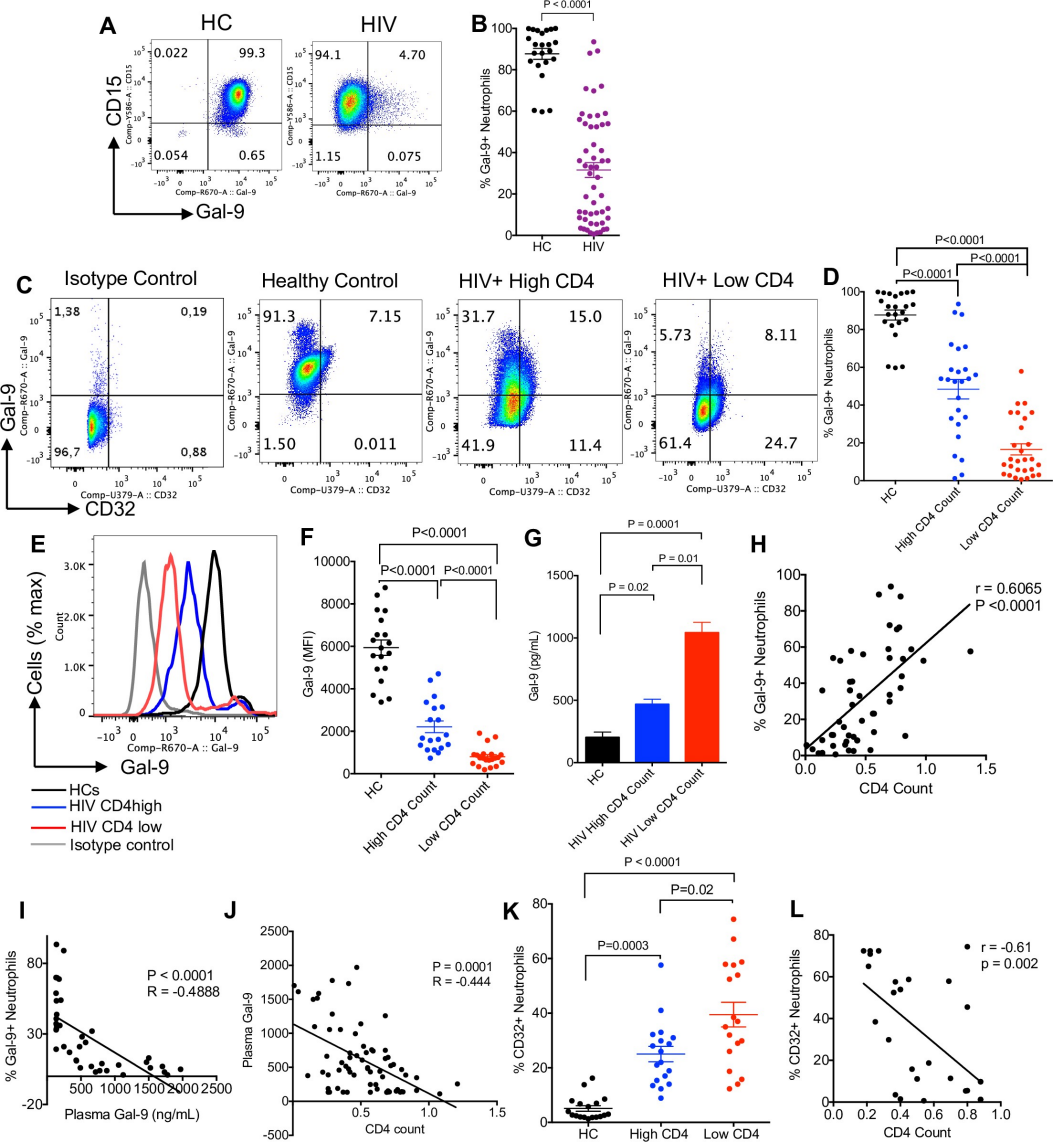
Neutrophils shed Gal-9 in HIV infection. **(A)** Representative plots and **(B)** cumulative data showing Gal-9 expression on neutrophils of HCs versus HIV-infected individuals. **(C)** Representative plots showing surface expression of Gal-9 and CD32 on neutrophils. **(D)** Cumulative data comparing surface expression of Gal-9 in neutrophils from HCs versus HIV patients with high and low CD4 T-cell count. **(E)** Representative histogram and **(F)** MFI of surface Gal-9 on neutrophils from HIV patients with high CD4 or low CD4 T-cell count and HCs. **(G)** ELISA results showing the concentration of soluble Gal-9 from unstimulated neutrophils obtained from HCs or HIV patients cultured for 8 hours. **(H)** Correlation between CD4 T-cell count and Gal-9+ neutrophils. **(I)** Correlation between Gal-9+ neutrophils and plasma Gal-9 in HIV patients. **(J)** Negative correlation between plasma Gal-9 and CD4 T-cell count in HIV patients. **(K)** Percentages of CD32-expressing neutrophils in HCs and HIV patients with high and low CD4 T-cell count. **(L)** Negative correlation between CD32 expression and CD4 T-cell count in HIV patients. The underlying data can be found in S1 Data. Gal-9, Galectin-9; HC, healthy control; MFI, median fluorescence intensity.

To evaluate how neutrophils from HIV-infected individuals with low CD4 T-cell count were enhancing T-cell activation, we speculated that neutrophils via soluble factors such as cytokines or cell–cell interactions may influence T-cell activation. To answer this question, we analyzed the transcriptional profile of cytokines that may modulate T-cell activation. Interestingly, no difference in the transcriptional expression of a variety of cytokines was noticed among the groups (**S4A Fig**). Next, we assessed the expression of co-inhibitory receptors, their ligands, and co-stimulatory molecules at the transcriptional levels. Although at the transcriptional levels some co-inhibitory/co-stimulatory molecules were highly expressed, and some had very low expression levels, we did not observe any significant difference in their expression in neutrophils of HIV patients regardless of their CD4 T-cell count with HCs (**S4B Fig**). To further investigate the potential role of these molecules in T-cell activation, the quantity of highly transcriptionally expressed genes was measured at the protein level by using flow cytometry. Using the gating strategy as depicted in **S4C Fig**, we did not find any significant differences in cell surface expression for HLA-DR, PDL-1/PDL-2, VISTA, and CD40 at the protein levels (**S5A–S5I Fig**). Similarly, no difference in the expression of CD48 and CD39 was noted. In contrast, we found a significant reduction in surface Gal-9 expression in neutrophils from HIV-infected individuals compared to HCs (**Fig 3A and 3B**). These observations suggested that differential Gal-9 expression on neutrophils may play a role in neutrophil-mediated T-cell activation. To address this question, we used a trans-well system to evaluate direct versus indirect effects of neutrophils on T-cell activation. These observations enabled us to conclude that a soluble factor is responsible for neutrophil-mediated T-cell activation (**S5J–S5M Fig**). Moreover, the addition of lactose (Gal-9 blocker) significantly abrogated the effects of neutrophil-mediated soluble factor in T-cell activation (**S5J–S5M Fig**).

### Surface Gal-9 expression on neutrophil decreases as HIV progresses

**Fig 4:**
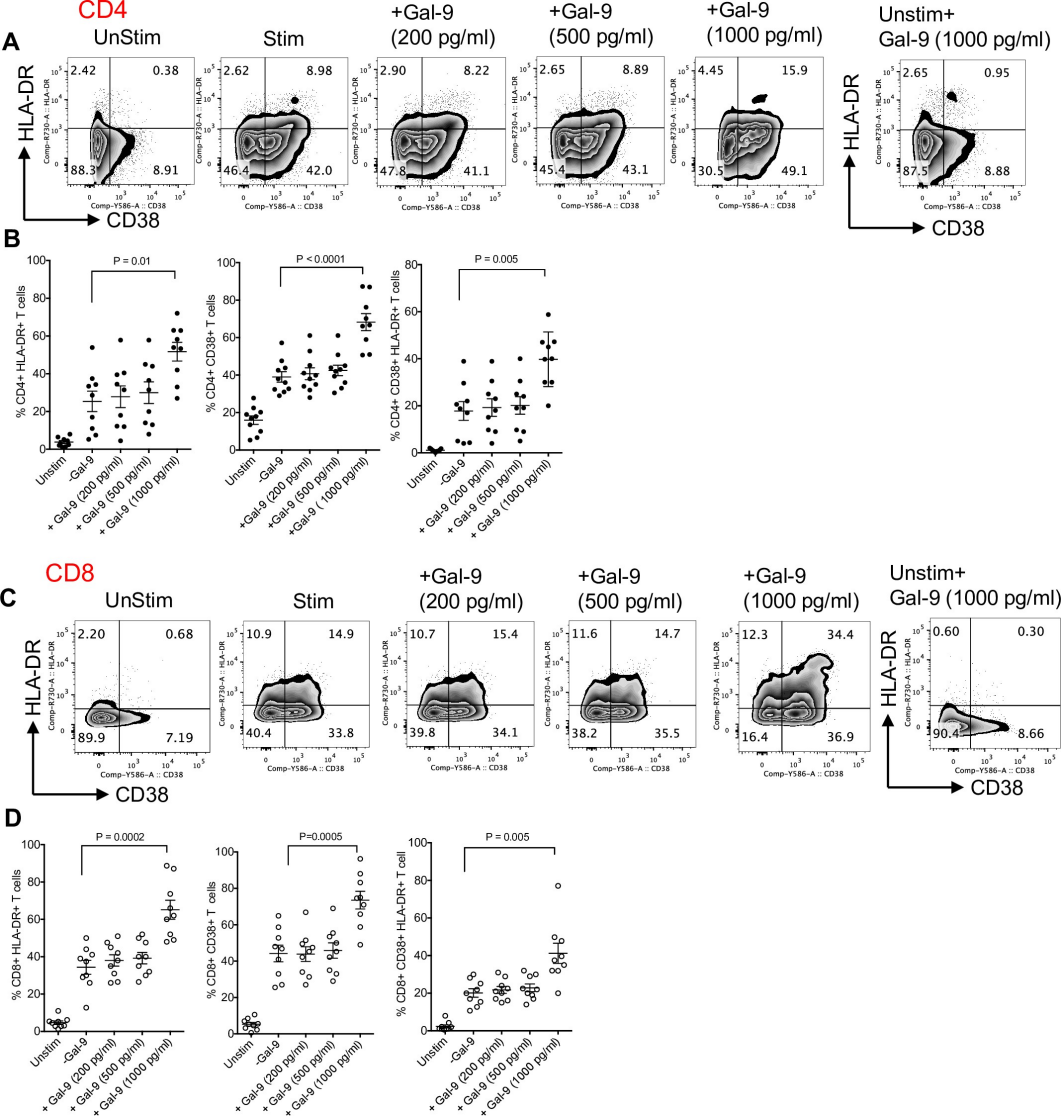
High concentrations of Gal-9 contribute to T-cell activation. **(A)** Representative flow cytometry plots and **(B)** cumulative data of percentages of CD4^+^ T cells expressing CD38, HLA-DR, and both when cultured and stimulated in the absence or presence of exogenous Gal-9 (200, 500, or 1,000 pg/ml) in vitro. **(C)** Representative flow cytometry plots and **(D)** cumulative data of percentages of CD8^+^ T cells expressing CD38, HLA-DR, and both when cultured and stimulated in the absence or presence of exogenous Gal-9 (200, 500, or 1,000 pg/ml) in vitro. The underlying data can be found in S1 Data. Gal-9, Galectin-9.

Upon observing the differential expression of Gal-9 on neutrophils from HCs versus HIV-infected individuals (**Figs 3A and 4B**), we aimed to further investigate the diversity of Gal-9 expression on neutrophils in HIV-infected individuals. We observed a significant decrease in the expression of surface Gal-9 on neutrophils in HIV patients as the disease progresses. As shown in **Fig 3C and 3D**, the decline in Gal-9 expression on neutrophils was more pronounced in HIV-infected individuals with low compared to high CD4 T-cell counts, and these groups had significantly lower levels of Gal-9 compared to HCs. This decline was not only significant in the percentages of Gal-9–expressing neutrophils but also in the intensity of Gal-9 expression (**Fig 3E and 3F**). These observations suggested that neutrophils from HIV-infected individuals may lose Gal-9 on their surface. To evaluate whether this Gal-9 was being shed or internalized, we measured the amount of soluble Gal-9 in the supernatant of unstimulated and cultured neutrophils (2 × 10^6^) for 8 hours. Interestingly, we found significantly higher levels of soluble Gal-9 in the culture supernatants of cultured neutrophils from HIV-infected individuals with low CD4 T-cell count compared to those with a higher CD4 T-cell count and HCs (**Fig 3G**). Additionally, we found a positive correlation between CD4 T-cell count and Gal-9–expressing neutrophils in HIV-infected individuals (**Fig 3H**). Moreover, an inverse correlation between the plasma Gal-9 concentrations and the percentages of Gal-9+ neutrophils was observed (**Fig 3I**). A similar pattern was observed for the plasma Gal-9 and CD4 T-cell count in HIV-infected individuals (**Fig 3J**). In contrast to the expression of Gal-9, we found that the expression of CD32 (FCγRIIA), which can be up-regulated by cell activation [59], was significantly higher in neutrophils of HIV-infected individuals, in particular, in those with lower CD4 T-cell count compared to HCs (**Fig 3K**). Further analysis confirmed a negative correlation between CD4 count with CD32 expression on neutrophils in HIV-infected individuals (**Fig 3L**). These observations suggest that activation status of neutrophils from HIV-infected individuals may result in Gal-9 shedding, which may play a role in HIV pathogenesis.

### Soluble Gal-9 intensifies T-cell activation

To evaluate the potential effects of secreted Gal-9 by neutrophils on T-cell activation, total PBMCs from HCs were stimulated with anti-CD3/CD28 in the presence or absence of different concentrations of recombinant Gal-9 in vitro. We found that recombinant Gal-9 at the concentration mimicking the detected level in cell culture supernatants of neutrophils from low CD4 T-cell count HIV patients (1,000 pg/ml) significantly enhanced T-cell activation as measured by the expression levels of CD38 and HLA-DR on both CD4^+^ and CD8^+^ T cells (**Fig 4A–4D**). However, Gal-9 at lower indicated concentrations did not significantly influence T cell activation (**Fig 4A–4D**). These observations suggest that neutrophils from HIV-infected individuals with low CD4 T-cell count shed higher concentrations of Gal-9, which explains their differential effects on T-cell activation in vitro. Of note, we did not observe any toxicity or increased cell death at the used concentrations of Gal-9 in vitro.

### Gal-9 enhances T-cell activation through CD44 binding and LCK signaling

**Fig 5:**
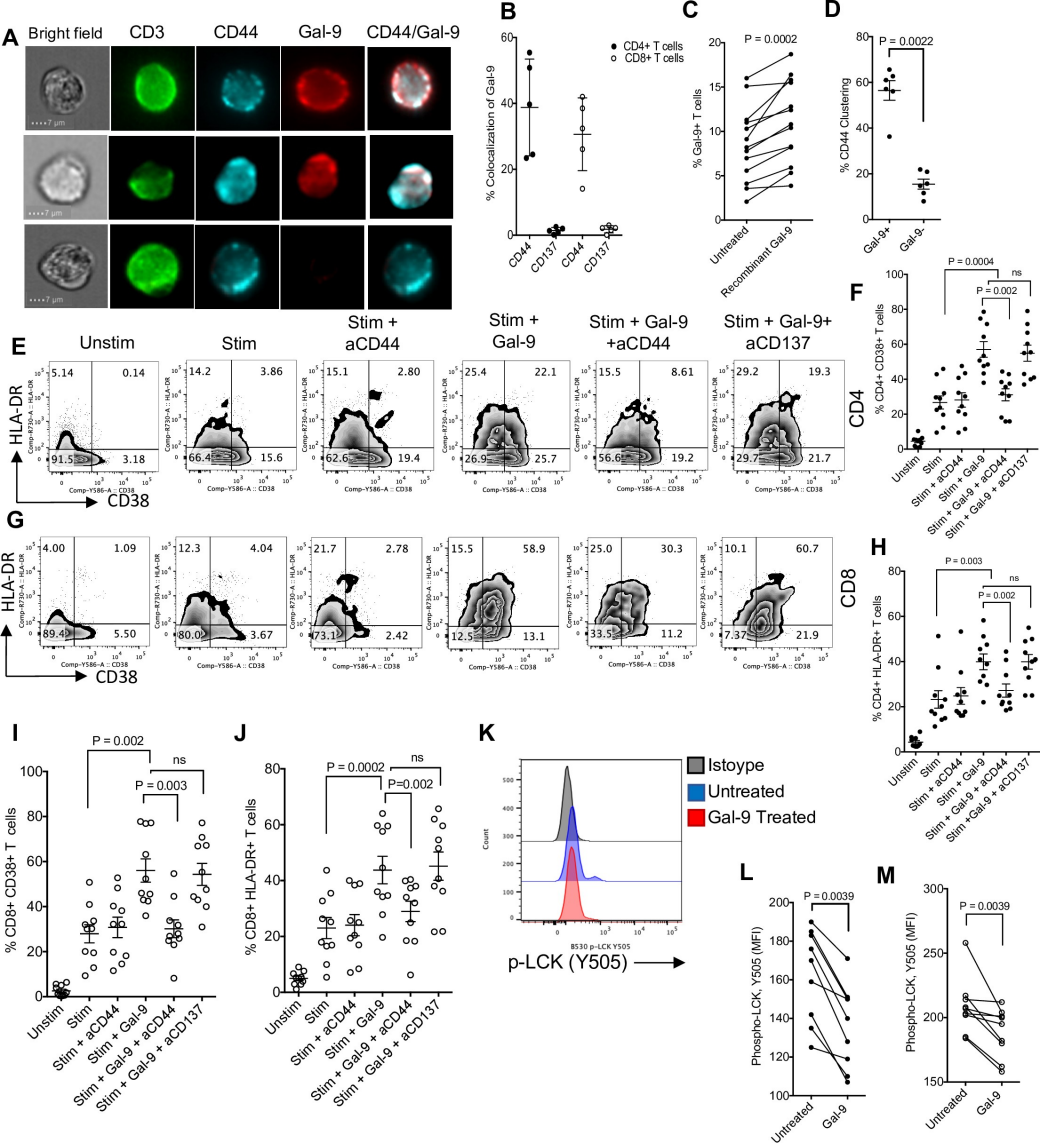
Gal-9 binds CD44 on T cells and enhances T-cell activation. **(A)** Images showing the expression of CD44 and Gal-9 on the surface of T cells. **(B)** Cumulative data showing percent colocalization of Gal-9 with CD44 and CD137. **(C)** Percent of Gal-9+ T cells in the presence or absence of recombinant Gal-9 following in vitro incubation. **(D)** Percent CD44 clustering in the presence and absence of Gal-9. **(E)** Representative plots and (**F**) cumulative data showing percentages of CD4^+^ T cells expressing CD38 or **(G)** HLA-DR following stimulation with anti-CD3/CD28 antibodies in the presence of exogenous Gal-9 (1,000 pg/ml) and/or Gal-9 and anti-CD44 and anti-CD137 antibodies. **(H)** Representative plots and (**I**) cumulative data showing percentages of CD8^+^ T cells expressing CD38 or **(J)** HLA-DR following stimulation with anti-CD3/CD28 antibodies in the presence of exogenous Gal-9 (1,000 pg/ml) and/or Gal-9 and anti-CD44 and anti-CD137 antibodies. **(K)** Representative plots and (**L**) cumulative data showing phospho-LCK (Y505) in CD4^+^ and **(M)** CD8^+^ T cells in the absence and presence of Gal-9 (1,000 pg/ml). The underlying data can be found in S1 Data. Gal-9, Galectin-9.

Although the interaction of plasma membrane Gal-9 on antigen-presenting cells (APCs) or Tregs with TIM-3 on T cells renders them to an exhausted phenotype [30,60,61], soluble Gal-9 enhances T-cell activation [62]. Thus, we decided to determine how soluble Gal-9 increases T-cell activation. We began by measuring the colocalization of surface Gal-9 expression on T cells with different markers that have been identified to interact/bind to Gal-9. We found that coculture of neutrophils from HIV-infected individuals with their PBMCs resulted in the colocalization of Gal-9 with CD44 on the surface of T cells (**Fig 5A and 5B**). However, this was not the case for other potential Gal-9 receptors such as CD137 (**Fig 5B**). To mimic these observations, T cells were incubated with 1,000 ng/mL of recombinant Gal-9 for 1 hour and then subjected to flow cytometry analysis. We found that the incubation of T cells with recombinant Gal-9 resulted in a significant increase in the percentages of Gal-9–expressing T cells (**Fig 5C**), suggesting the binding of recombinant Gal-9 to T cells. Subsequently, Gal-9 binding to T cells increased CD44 clustering on T cells (**Fig 5D**). These observations indicated that Gal-9 interacts with CD44 on T cells. To determine whether such interactions result in T-cell activation, we examined the commonly used T-cell activation markers in HIV infection, CD38 and HLA-DR, following stimulation with anti-CD3/CD28 antibodies in the presence or absence of recombinant Gal-9 and anti-CD44 or anti-CD137 blocking antibodies for 48 hours. These studies confirmed that the recombinant Gal-9 enhances T-cell activation as shown by the up-regulation of CD38 and HLA-DR; however, anti-CD44 blocking antibody significantly prevents such effect (**Fig 5E–5J**). In contrast, the anti-CD137 antibody did not reduce T-cell activation (**Fig 5E–5J**). These observations suggest that recombinant Gal-9 via interactions by CD44 enhances T-cell activation. Importantly, we found that this occurs through LCK signaling as recombinant Gal-9 (1,000 pg/ml) decreases phospho-LCK (Y505) intensity in T cells (**Fig 5K–5M**) in both CD4^+^ and CD8^+^ T cells, respectively. In agreement, it is shown that pretreatment of T cells with LCK inhibitor, which suppresses LCK phosphorylation on Tyr-505, abrogates stimulatory effects of Gal-9 on T cells possibly through calcium mobilization [62]. These results indicate that exogenous Gal-9 interaction with CD44 contributes to T-cell activation.

### Neutrophils shed Gal-9 by CD44 depalmitoylation

**Fig 6:**
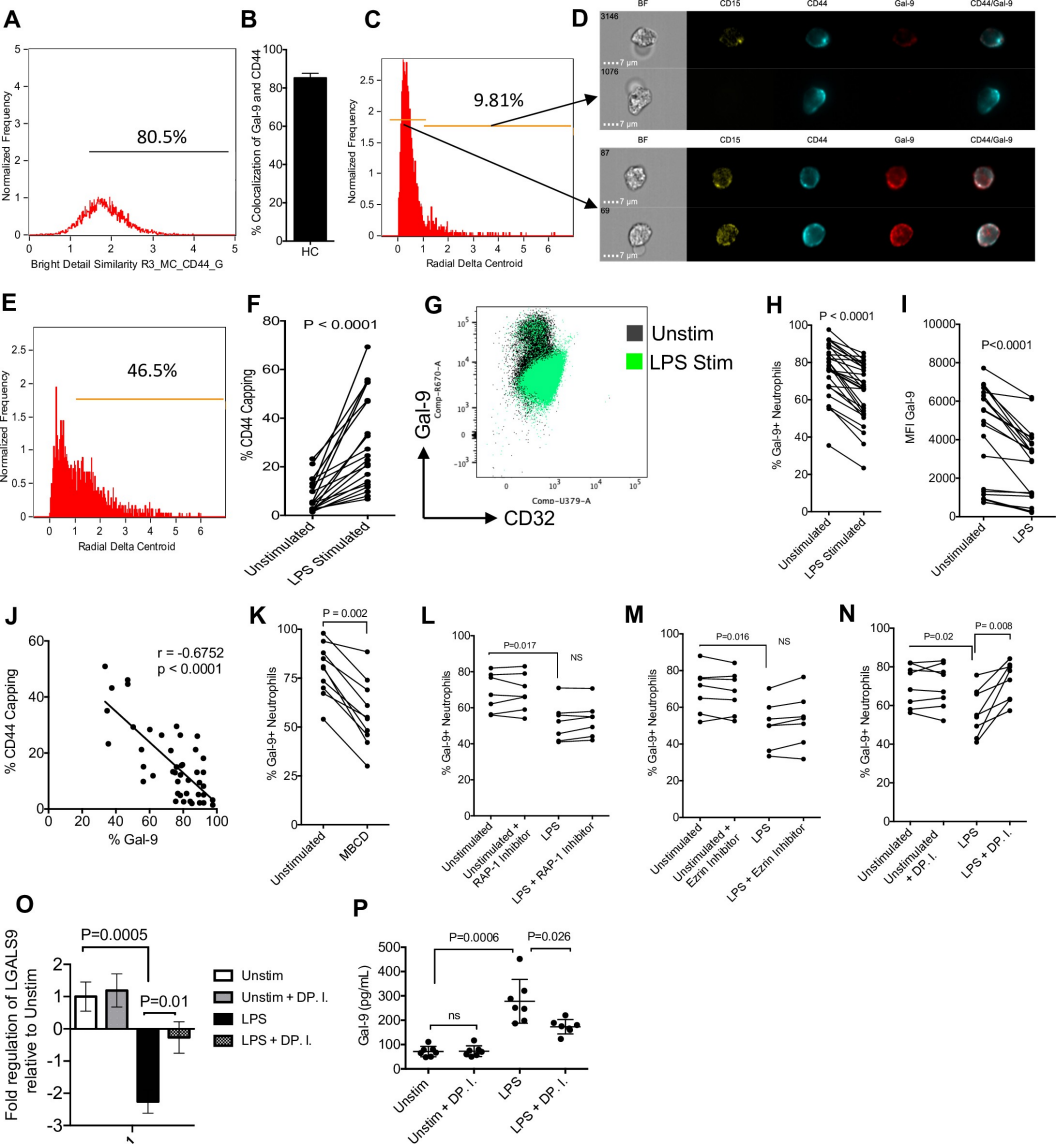
Gal-9 is shed by neutrophil mediated DP of CD44 upon activation. **(A)** Percent colocalization of CD44 and Gal-9 on neutrophils quantified using an Amnis ImageStream. **(B)** Cumulative percent colocalization of Gal-9 and CD44 on the surface of neutrophils. **(C)** Representative plot of neutrophils with a high and low delta centroid XY. **(D)** Representative images of capped and dispersed CD44 on neutrophils. **(E)** Representative plot of LPS-treated neutrophil radial delta centroid. **(F)** Cumulative data of CD44 capping (delta centroid >1) in unstimulated and LPS-treated neutrophils. **(G)** Representative plot showing changing Gal-9 and CD32 expression on neutrophils untreated or stimulated with LPS. **(H)** Cumulative results showing the percent expression of surface Gal-9 on unstimulated and LPS stimulated neutrophils. **(I)** Cumulative results showing the MFI of surface Gal-9 on unstimulated and LPS stimulated neutrophils. **(J)** Cumulative data showing correlation between % Gal-9 expression on neutrophils and CD44 capping. **(K)** Surface expression of Gal-9 on neutrophils untreated or treated with MBCD. **(L)** Surface expression of Gal-9 on neutrophils untreated or treated with LPS in the presence or absence of a RAP-1 inhibitor, **(M)** ezrin inhibitor, and a **(N)** DP.I. **(O)** Cumulative data of Gal-9 mRNA expression and (**P**) shed Gal-9 in culture supernatants of LPS-activated neutrophils in neutrophils from HCs once stimulated with LPS for 3 hours in the presence or absence of DP.I. The underlying data can be found in S1 Data. DP, depalmitoylation; DP.I, depalmitoylation inhibitor; Gal-9, Galectin-9; HC, healthy control; LPS, lipopolysaccharide; MBCD, methyl-beta-cyclodextran; MFI, median fluorescence intensity.

Upon observing that Gal-9 shedding occurs in neutrophils of HIV-infected individuals, we decided to mechanistically explore how neutrophils shed Gal-9. We observed that Gal-9 colocalizes with CD44 on the surface of neutrophils even at greater extent compared to T cells (**Fig 6A and 6B**). Thus, we utilized imaging cytometry to analyze the organization of CD44 on neutrophils by the gating strategy detailed in **S6A Fig**. We observed that when CD44 capping occurs (high radial delta centroid), Gal-9 diminishes from the surface of neutrophils (**Fig 6C and 6D**). Moreover, we found that stimulation of neutrophils by lipopolysaccharide (LPS) induces (1 hour) CD44 capping (**Fig 6E and 6F**). Subsequently, stimulation of neutrophils by LPS significantly reduced the surface expression of Gal-9 (**Fig 6G–6I**, **S6B Fig**). Based on these observations, we found a reverse correlation between CD44 capping and % Gal-9–expressing neutrophils (**Fig 6J**). To further evaluate whether the movement of CD44 from the lipid raft contributes to the Gal-9 shedding, we exposed neutrophils to 30 mM of methyl-beta-cyclodextran (MBCD) for 20 minutes (depleting lipid rafts). Upon depletion of lipid rafts, we observed a decrease in the expression of Gal-9 (**Fig 6K**). Upon release from the lipid raft, CD44 has been shown to interact with the actin cytoskeleton through an interaction with the molecules RAP-1 and ezrin [63]. We decided to determine if the inhibition of ezrin and RAP-1 can prevent Gal-9 shedding. However, our observations indicated that Ezrin and RAP-1 inhibitors did not inhibit Gal-9 shedding following stimulation of neutrophils by LPS (**Fig 6L and 6M**). It has been reported that to facilitate the movement of CD44 from the lipid rafts, DP enzymes will depalmitoylate lipid raft associated CD44 [16]. We found that the processes of DP contributes to the CD44 capping and shedding of Gal-9 on neutrophils (**Fig 6N**). To gain a better understanding of this process, we measured the expression of Gal-9 mRNA in activated neutrophils and soluble Gal-9 in the culture supernatant after 3-hour stimulation with LPS. We found that LPS stimulation significantly down-regulated Gal-9 mRNA but increased soluble Gal-9 in the culture supernatant (**Fig 6O and 6P**). The inhibition of DP partially reversed the down-regulation Gal-9 mRNA by LPS and its shedding from neutrophils (**Fig 6O and 6P**). These results indicate that the movement of CD44 from the lipid raft, and, more specifically, the DP of CD44 induces the shedding of Gal-9 from neutrophils.

### Glycolysis and ROS production regulates CD44 polarization and Gal-9 shedding in neutrophils

**Fig 7:**
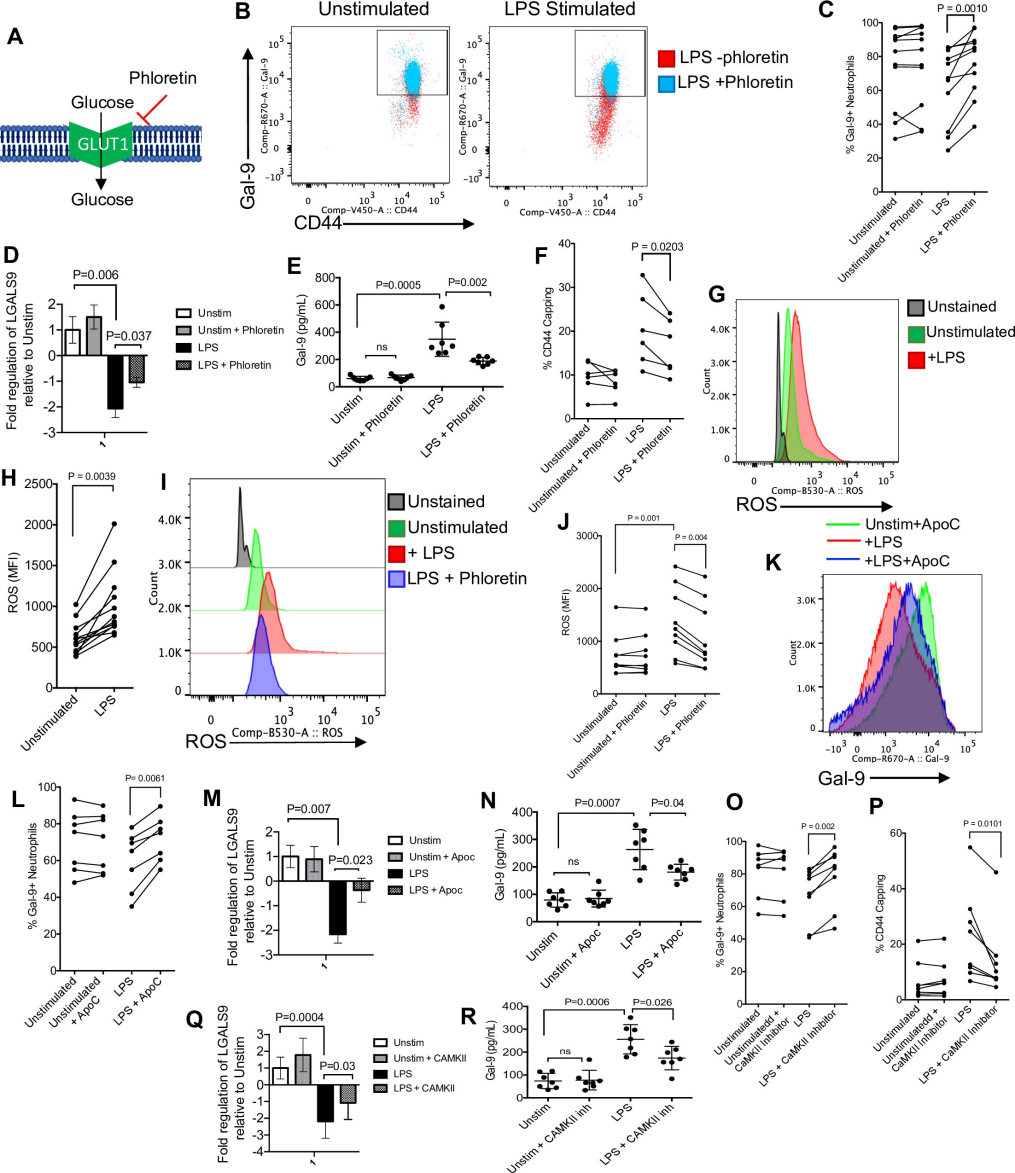
Gal-9 shedding by activated neutrophils is regulated by ROS and CaMKII. **(A)** Visual model showing the mechanistic action of phloretin on the GLUT1 transporter. **(B)** Representative plots showing the effect of phloretin on Gal-9 shedding. **(C)** Cumulative data showing surface Gal-9 expression in untreated or LPS-treated neutrophil in the presence or absence of phloretin. **(D)** Cumulative data of Gal-9 mRNA expression and **(E)** shed Gal-9 in culture supernatants of LPS-activated neutrophils in neutrophils from HCs once stimulated with LPS for 3 hours in the presence or absence of phloretin. **(F)** Cumulative data showing percent CD44 capping in the presence or absence of phloretin in untreated or LPS-treated neutrophils. **(G)** Representative plots and **(H)** cumulative data representing the MFI of ROS in untreated and LPS-treated neutrophils. **(I)** Representative plots and **(J)** cumulative data showing the change in MFI of ROS expression in the presence or absence of LPS and phloretin. **(K)** Representative histogram and **(L)** cumulative data showing percent Gal-9 expression in the absence and presence of LPS and LPS + Apoc. **(M)** Cumulative data of Gal-9 mRNA expression and **(N)** shed Gal-9 in culture supernatants of LPS-activated neutrophils in neutrophils from HCs once stimulated with LPS for 3 hours in the presence or absence of Apoc. **(O)** Cumulative data showing percent Gal-9 expression and **(P)** CD44 capping in the absence or presence of LPS and a CaMKII inhibitor. **(Q)** Cumulative data of Gal-9 mRNA expression and **(R)** shed Gal-9 in culture supernatants of LPS-activated neutrophils in neutrophils from HCs once stimulated with LPS for 3 hours in the presence or absence of CAMKII inhibitor. The underlying data can be found in S1 Data. Apoc, Apocynin; CaMKII, calcium/calmodulin-dependent protein kinase II; Gal-9, Galectin-9; HC, healthy control; LPS, lipopolysaccharide; MFI, median fluorescence intensity; ROS, reactive oxygen species.

Upon mechanistically identifying how Gal-9 is shed from neutrophils during activation, we aimed to investigate the regulatory process contributing to the Gal-9 shedding. By utilizing the small molecule inhibitor of glucose transporter 1 (GLUT1), phloretin, we observed an inhibition of Gal-9 shedding in LPS-stimulated neutrophils (**Fig 7A–7C**). We also found that LPS activation significantly reduced the expression of Gal-9 mRNA and partially prevented its shedding from activated neutrophils (**Fig 7D and 7E**). Although neutrophil activation increases CD44 capping (**Fig 6E and 6F**), this CD44 capping was impaired by phloretin (**Fig 7F**). In addition, we observed that stimulation of neutrophils with LPS resulted in a significant increase in the expression of ROS (**Fig 7G and 7H**). To determine whether phloretin can impact ROS production by neutrophils, we stimulated neutrophils with LPS in the presence or absence of 100 nM phloretin for 1 hour. We found that phloretin significantly inhibited ROS expression by neutrophils (**Fig 7I and 7J**). In order to better understand the role of ROS in Gal-9 shedding, we treated neutrophils with LPS in the presence or absence of Apocynin (Apoc) according to our previous report [53]. As shown in **Fig 7K and 7L**, as predicted, LPS stimulation significantly reduced surface Gal-9 expression, but the inhibition of ROS with Apoc treatment restored Gal-9 levels on neutrophils. As shown in **Fig 7D**, we observed that LPS-mediated down-regulation of Gal-9 was at the gene level. However, Apoc partially inhibited LPS-induced Gal-9 mRNA down-regulation and its shedding from neutrophils as we detected lower soluble Gal-9 in the culture supernatant (**Fig 7M and 7N**). Knowing that ROS oxidizes CaMKII [64], we evaluated whether Gal-9 shedding can be prevented by treating activated neutrophils with a CaMKII inhibitor. Remarkably, we found that although LPS stimulation sheds Gal-9 from the surface of neutrophils, treatment with a CamKII inhibitor prevented Gal-9 shedding (**Fig 7O**) and subsequently reduced CD44 capping (**Fig 7P**). Finally, we found that the inhibition of CamKII partially abrogated LPS-mediated Gal-9 down-regulation and subsequently reduced its shedding (**Fig 7Q and 7R**). Taken together, these results demonstrate that glucose metabolism, ROS production, and CamKII activation regulate neutrophil shedding of Gal-9.

### Neutrophil shedding of Gal-9 is regulated by IL-10 in HIV infection

**Fig 8:**
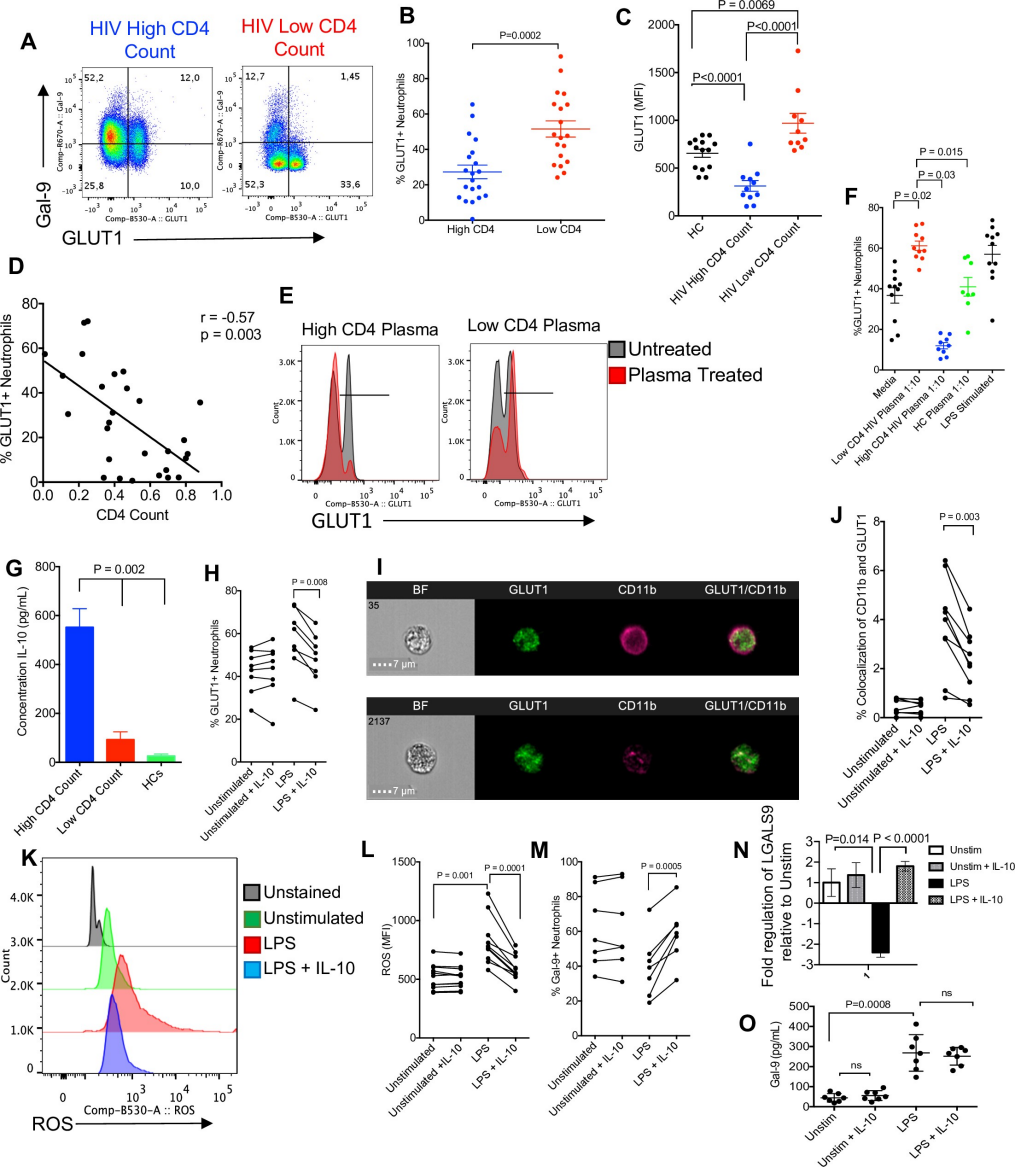
Neutrophils from HIV-infected individuals with high CD4 T-cell count have increased IL-10 activity and decreased glycolysis. **(A)** Representative plots and **(B)** cumulative data of GLUT1 and Gal-9 expression in neutrophils of HCs versus HIV patients with low or high CD4 T-cell count. **(C)** Cumulative data showing the MFI of GLUT1 in neutrophils from HCs versus HIV patients with low or high CD4 T-cell count. **(D)** Correlation of GLUT1 expression and CD4 T-cell count on neutrophils of HIV-infected patients. **(E)** Representative plots and **(F)** cumulative data of GLUT1 expression in neutrophils treated or untreated with plasma from HCs, HIV patients with low and high CD4 T-cell count. **(G)** ELISA results showing IL-10 concentrations in the plasma of HIV-infected individuals with low and high CD4 T-cell count and HCs. **(H)** GLUT1 expression in the presence or absence of IL-10 treatment and/or LPS. **(I)** Representative images of GLUT1 and CD11b. **(J)** Cumulative data showing colocalization of CD11b and GLUT1 in the presence or absence of IL-10 and/or LPS. **(K)** Representative plots and **(L)** cumulative data of ROS expression on neutrophils in the presence of IL-10 and LPS. **(M)** Cumulative data of Gal-9 expression on neutrophils in the presence or absence of IL-10 and/or LPS. **(N)** Cumulative data of Gal-9 mRNA expression and (**O**) shed Gal-9 in culture supernatants of LPS-activated neutrophils in neutrophils from HCs once stimulated with LPS for 3 hours in the presence or absence of IL-10 (400 pg/ml). The underlying data can be found in S1 Data. Gal-9, Galectin-9; HC, healthy control; IL, interleukin; LPS, lipopolysaccharide; MFI, median fluorescence intensity; ROS, reactive oxygen species.

We wanted to evaluate what regulatory mechanism(s) could explain the differences in Gal-9 expression on neutrophils from HIV-infected patients with high CD4 T-cell count versus those with low CD4 T-cell count. As we showed, ROS induces the shedding of Gal-9, and blocking ROS synthesis through glycolysis is effective to sustain Gal-9 surface expression. Since GLUT1 plays an essential role in the regulation of ROS [65], we measured the surface expression of Gal-9 on neutrophils and found that HIV patients with high CD4 T-cell count had lower percentages of GLUT1-expressing neutrophils and also lower intensity of GLUT1 (**Fig 8A–8C**). In addition, we found an inverse correlation between percentages of GLUT1+ neutrophils and CD4 T-cell count in HIV-infected individuals (**Fig 8D**). To evaluate whether GLUT1 expression is modulated by a plasma soluble factor, plasma specimens (10% plasma in culture media for 1 hour) from HIV patients with low CD4 T-cell count or high CD4 T-cell count were added to unstimulated neutrophils of HCs. We observed that GLUT1 expression was significantly reduced when neutrophils were treated with plasma from HIV patients with high CD4 T-cell count but not from those with low CD4 T-cell count (**Fig 8E**). Interestingly, the plasma from HIV-infected individuals with low CD4 T-cell count significantly increased GLUT1 expression, whereas the opposite effect was observed by the plasma from HIV-infected individuals with high CD4 T-cell count (**Fig 8F**). However, plasma from HCs did not change GLUT1 expression, but treatment of neutrophils with LPS significantly increased GLUT1 expression (**Fig 8F**). To better understand the underlying mechanism of differential effects of plasma on GLUT1 expression, the plasma of HIV-infected individuals was subjected to cytokine ELISAs. Among the detected cytokines in the plasma (tumor necrosis factor alpha [TNFα], interleukin (IL)-4, IL-10, transforming growth factor beta [TGFβ], IL-8, IL-6, IL-7, and IL-13), we noticed that HIV patients with high CD4 T-cell count had substantially elevated concentrations of IL-10 compared to the plasma from HIV patients with low CD4 T-cell count and HCs (**Fig 8G**). In agreement with previous studies, the IL-10 responding gene DDIT4 was up-regulated in neutrophils from HIV-infected patients with high CD4 count (**S6C Fig**) [66]. To better understand the role of IL-10 on GLUT1 expression in neutrophils, we cultured neutrophils in the presence of recombinant IL-10 (400 pg/ml) either unstimulated or stimulated with LPS. As predicated, LPS up-regulated GLUT1 expression on neutrophils; however, addition of IL-10 abrogated the LPS effects and significantly reduced GLUT1 expression (**Fig 8H**). This agrees with previous reports that IL-10 decreases GLUT1 translocation [66]. Using the same approach, we found that GLUT1 translocation was inhibited by IL-10 (**Fig 8I and 8J**). Moreover, we observed that although LPS enhanced ROS production in neutrophils, IL-10 reversed this effect and significantly reduced ROS production by activated neutrophils (F**ig 8K and 8L**). Consequentially, Gal-9 shedding was inhibited by IL-10 treatment (**Fig 8M**). This was further confirmed by the inhibitory role of IL-10 on LPS-induced Gal-9 mRNA down-regulation and its shedding in culture supernatants (**Fig 8N and 8O**). These observations suggest that Gal-9 shedding in HIV patients with high CD4 T-cell count could be inhibited by the presence of IL-10.

## Discussion

In this study, we found that neutrophils in HIV-infected individuals have an activated phenotype and shed Gal-9, which contributes to T-cell function by a newly identified mechanism.

This is in agreement with previous studies that have shown that phenotypically neutrophils are activated and have increased adhesion marker expression and ROS production in HIV-infected individuals [25,26,67]. We found that neutrophils in HIV infection have a transcriptional profile that is associated with an activated phenotype in comparison to neutrophils from HCs. Previous work has suggested that neutrophil activation in HIV infection is mediated through IL-18, IL-17, and IL-8 signaling [67]. Despite these important observations, previous studies have not distinguished which are contributing to neutrophil activation. Our data indicate that neutrophils have increased expression of specific genes associated with the p38 MAPK cascade, NFAT signaling, and FcγR signaling.

Through the analysis of transcriptional profiles of HIV-infected individuals, we found that neutrophils from HIV patients with low CD4 T-cell counts have increased expression of genes associated with a variety of signaling activities. These observations are in agreement with previous studies that have shown that neutrophils from HIV patients with low CD4 T-cell counts were more activated than those with high CD4 T-cell counts [25]. These results indicate a change in neutrophil biology in HIV patients as the disease progresses. One possible explanation for the differential neutrophil activation status might be because of the increased bacterial product translocation from the gut [68].

During HIV infection, T-cell activation can be associated with HIV disease progression, and, therefore, increased T-cell activation could be promoting HIV pathogenesis [69]. Previous studies have attributed neutrophil function to the activation and/or suppression of T cells during HIV infection [35,70]. Our study suggests that neutrophils are capable of enhancing T-cell activation if they are from HIV patients with low CD4 T-cell count only. These observations suggest that neutrophils are not created equally in all HIV-infected individuals.

During viral infections, neutrophils express HLA-DR (MHC class II), CD80, and CD40, which contribute to antigen-specific CD4^+^ T-cell response [71]. However, the expression of HLA-DR, CD80, and CD40 on neutrophils requires antigen exposure and a long-term neutrophil cell culture (6 days) which may not be physiologically relevant as neutrophils have a short half-life. In another study, the expression of HLA-DR has been shown to be expressed on the surface of neutrophils from patients treated with recombinant interferon gamma (IFNγ) (44 hours) [72–74]. Despite these reports, our studies did not find HLA-DR expression on the surface of neutrophils.

We also found that neutrophils in HIV infection do not express substantial levels of PD-L1, PD-L2, TIM-3, and VISTA. Initially, these observations may appear as being inconsistent with a study by Bowers and colleagues, but this study had evaluated the suppression of T cells through PD-L1 on LDW neutrophils [35], while our study only focused on normal density mature neutrophils. These LDW neutrophils are considered immature and are functionally unique in comparison to mature neutrophils [75]. LDW express PD-L1, which can suppress T-cell function via PD-1:PDL-1in HIV infection [35]. However, based on our data, the expression of PDL-1 was negligible in mature neutrophils.

Interestingly, neutrophil cytokine expression and co-inhibitory/co-activation receptors expression did change between HIV infected or HCs; however, we found that Gal-9 protein surface expression was reduced on neutrophils from HIV patients. This observation led us to further investigate the mechanism underlying Gal-9 down-regulation from the surface of neutrophils.

As neutrophils become activated in HIV-infected individuals, we speculated that Gal-9 gets cleaved or shed. Interestingly, we observed that the activation of neutrophils from HCs in vitro down-regulated Gal-9 at the gene level and increased its shedding. However, despite the activated status of neutrophils in the context of HIV infection, we did not observe any significant difference between the Gal-9 expression at the gene level between the groups. It is worth mentioning that an acute LPS activation in vitro may not reflect the chronicity of HIV infection and the influence of other soluble factors and mediators in vivo. The plasma soluble Gal-9 level in a variety of pathological conditions has been shown to increase [76]. For example, in acute HIV infection, plasma concentrations of Gal-9 are increased similar to TNFα and IFNα [41]. A similar observation has been made for the plasma Gal-9 and pro-inflammatory cytokines in Severe Acute Respiratory Syndrome Coronavirus 2 (SARS-CoV-2)–infected individuals [77]. In agreement, our findings show that the activity of neutrophils from HIV patients is correlated with the level of exogenous Gal-9 in the plasma. Neutrophils from HIV-infected patients with low CD4 cell count are capable of shedding substantial quantities of Gal-9, and the surface Gal-9 expression of neutrophils negatively correlates with its plasma levels. While it is difficult to claim that neutrophils are the only major source of soluble Gal-9 in the plasma because of diverse tissue expression of Gal-9 in the body [37], based on our data, neutrophils are a potent source of soluble Gal-9 in vitro as reported in SARS-CoV-2–infected individuals [77].

Soluble Gal-9 has been associated with T-cell activation through ERK signaling and LCK activation [44,62]. In agreement, we found that soluble Gal-9 is capable of enhancing the activation of T cells at >1,000 pg/mL, but not at lower concentrations. Previous in vivo findings show that Gal-9 activates T cells in a dose-dependent manner [78]. It is important to distinguish biological properties of the membrane bound Gal-9 on T cells [49,79], which is reported to induce T-cell exhaustion versus the soluble/secreted form of Gal-9.

We also found that Gal-9 is capable of binding to CD44 on the cell surface, which causes the stabilization of dephosphorylated LCK and enhancement of T-cell activation. We observed an increase in CD44 clustering on T cells in the presence of Gal-9. Previous studies have shown that CD44 clustering and cross-linking (by antibodies) can impact T-cell signaling through the stabilization of LCK activation and protein kinase C (PKC)-mediated movement [80,81].

LCK is an integral T-cell signaling molecule that promotes intracellular signaling during T-cell activation. LCK can be phosphorylated at the tyrosine 394 residue (the activating phosphorylation site) and the tyrosine 505 residue (inactivation phosphorylation site). CD45 dephosphorylates CD44 at both the Y505 and Y395 residue, which can both positively or negatively impact T-cell activation [82]. Interestingly, CD45 is restricted from the lipid raft in inactive T cells and contributes to ERK activation [83]. As a result of CD45 exclusion from lipid rafts, CD44 binds LCK and remains in the lipid rafts of T cells, which contributes to the stabilization of dephosphorylated (primed) or activated LCK. This stabilization of LCK in lipid rafts can be enhanced by the cross-linking of CD44. Our results suggest that Gal-9 can contribute to the clustering of CD44 in T cells, which acts as a biological cross-linking factor of CD44 and contributes to the stabilization of dephosphorylated/active LCK.

Our observations showed that Gal-9 promotes T-cell activation and that T-cell stimulation is reduced by the inhibition of CD44 [62,84]. Gal-9 can contribute to ERK signaling and consequentially increases transcription of latent HIV in vitro [43,44]. Taking these results into consideration, it is difficult to interpret whether Gal-9 is detrimental to HIV infection or beneficial. Our study shows that Gal-9 treatment increases T-cell activation markers. On the other hand, we have already shown that the interaction of exogenous Gal-9 with TIM-3 on CD4^+^ T cells reduces HIV infection/replication but its interaction with PDI enhances HIV infection [36]. In contrast, LCK activity has been associated with differentially modulating CD8^+^ T-cell polarization and could be protective in CD8^+^ T-cell–mediated immune responses [85,86]. CD8^+^ effector memory T cells have more LCK in the active conformation, which suggests that increased LCK signaling could produce a more robust and functional CD8^+^ T-cell response [85]. Thus, increased Gal-9 signaling in T cells from HIV patients can either increase or decrease viral replication depending on the interacting ligand target (e.g., PDI or TIM-3) [36,39]. However, enhanced CD8^+^ T-cell activation may be a double-edged sword. Activated CD8^+^ T cells by eliminating virally infected cells can play a protective role; however, prolonged immune activation can drive T-cell exhaustion and chronic hyperimmune activation in HIV-infected individuals.

In light of our observations, we found that neutrophils regulate the shedding of Gal-9 by cytoskeletal remodeling of surface CD44 expression through a regulated process. The movement of CD44 in neutrophils is a regulated process that facilitates the binding of CD44 to hyaluronan, an integral step in the recruitment of neutrophils to a variety of tissues [15]. Specifically, this process is essential for neutrophil extravasation into the sinusoids of the liver and is implicated to the movement into other tissues [2,14,18,19]. Our study identified that neutrophil polarization is linked to the induction of ROS, calcium release, and the engagement of CD44 with the actin cytoskeleton.

The binding of Gal-9 to neutrophils has been implicated to reduce neutrophil recruitment to the lungs and suggests a potential need for neutrophils to shed surface-bound Gal-9 for optimal recruitment to the inflamed tissue in an allergic mouse model [87]. However, Gal-9 is not normally bound to CD44 on murine neutrophils under homeostatic conditions, and, therefore, we cannot assume that these interactions will be reproducible in the human system (**S6D–S6F Fig**). Although we have no evidence to suggest that the increased Gal-9 binding on human neutrophils is capable of suppressing the biochemical movement of CD44 out of lipid rafts, we revealed that through an indirect manner, neutrophils regulate and promote the shedding of Gal-9 through the cytoskeletal reorientation of CD44.

When activated, neutrophils depalmitoylate CD44 and promote its interaction with ezrin to move CD44 to one end of the neutrophil [20]. DP facilitates the movement of CD44 out of the lipid raft, where CD44 can interact with ezrin and the actin cytoskeleton [16]. We found that the DP of CD44 during neutrophil activation facilitates the movement of CD44, and, subsequently, Gal-9 shedding. The process of shedding Gal-9 is regulated specifically by the DP of CD44 but not the interaction with the cytoskeleton (binding ezrin/RAP-1).

Neutrophil activation is primarily fueled by glycolysis metabolism, and our study identified that Gal-9 shedding is dependent on the activity of glycolysis [88]. Glycolysis fuels a regulatory mechanism that is essential for Gal-9 shedding. Additionally, GLUT1 is a prominent glucose transporter in neutrophils and contributes significantly to the function of neutrophils [88]. To further evaluate the impact of glycolysis on neutrophil activity, we measured ROS production following neutrophil stimulation with LPS and/or phloretin and found that the inhibition of GLUT1 impaired the production of ROS. Neutrophils use glycolysis to fuel the pentose–pyruvate phosphatase pathway (PPP) to generate NADPH. NADPH will then be used to fuel NOX2, a ROS-producing enzyme that is essential in immune protection facilitated by neutrophils [6]. This process appears to be essential for Gal-9 shedding as our observations indicated that ROS and glycolysis are important mediators in the DP of CD44. Interestingly, we found that if ROS production or activity is inhibited (by a ROS scavenger), Gal-9 release can be prevented, therefore suggesting an association between Gal-9 shedding, neutrophil metabolism, and ROS production. From these observations, we identified that neutrophil shedding of Gal-9 is regulated by CaMKII.

CaMKII is a calcium/ROS-activated kinase that phosphorylates CD44 at the Ser325 residue, which causes a conformational change in CD44 allowing of DP [2,16,64]. As a result of CaMKII regulating CD44 DP, we showed that CaMKII is responsible for the regulation of Gal-9 shedding. This agrees with a previous study by Lewis and colleagues showing that Ser325 phosphorylation of CD44 is required for binding to hyaluronan [13], which indicates an integral role for CaMKII activation in CD44-mediated extravasation. CaMKII is an essential regulatory element of Gal-9 shedding because the DP enzymes LYPLA1, LYPLA2, PPT1, ABHD12, ABHD17A, and ABHD17B are expressed by neutrophils (**S7A Fig**) [89]. This biochemical observation suggests that the conformational changes in the induction of CD44 by CaMKII are the primary mechanisms to promote CD44 DP.

Interestingly, CaMKII can be activated by ROS, and, therefore, helps to explain our observations that glycolysis and ROS production promote neutrophil shedding of Gal-9 in response to activation. Our model suggests that during HIV infection, neutrophils are activated and produce ROS through NOX2 activity. The release of ROS from neutrophils in HIV infection becomes H_2_O_2_, which can pass back through the cell membrane of the neutrophils to activate CaMKII. This mechanism is reflected with increased ROS circulating in HIV patients [67,90]. Although these observations provide important information regarding the processes that contribute to Gal-9 shedding by neutrophils in HIV infection, they do not explain why neutrophils from HIV patients with low CD4 T-cell count have more prominent Gal-9 shedding compared to patients with high CD4 T-cell count despite their similar activation transcriptional profile.

These discrepancies lead us to investigate the potential role of ROS/glycolysis-modifying proteins that are present in HIV infection. Myeloid cell activation and metabolism has previously been shown to be modulated by IL-10 signaling [66]. During macrophage activation, IL-10 signals through the IL-10 receptor, causing the increase of DDIT4 expression and inhibition of secretory vesicle translocation, consequentially reducing the surface expression of GLUT1 [66]. Our RNA-seq analysis revealed a higher DDIT4 expression in neutrophils of HIV patients with high CD4 T-cell count. Furthermore, genes associated with glycolysis were highly expressed in HIV-infected patients with low CD4 T-cell count in comparison to HIV patients with high CD4 count or HCs (**S7B Fig**). Additionally, we observed lower surface expression of GLUT1 on the surface of neutrophils from patients with high CD4 T-cell count. These results suggest that neutrophils are similarly impacted by IL-10 in HIV infection. In the presence of plasma from HIV patients with high CD4 T-cell count and recombinant human IL-10, a reduction in GLUT1 on the surface of neutrophils was observed. Thus, ROS and glycolysis in HIV-infected patients contribute to Gal-9 shedding through the activation of CaMKII.

We are aware of our study limitations. For example, the type of ART treatment, age, sex, viral load, and the duration of viremia in HIV patients may influence the biological properties of neutrophils. However, we were unable to obtain and consider these confounding factors in our analysis. We are also aware that other immune and nonimmune cells may shed Gal-9. Besides, we were unable to measure cytokine production capabilities of neutrophils in different groups. Moreover, we were unable to determine whether viral load can influence Gal-9 shedding from neutrophils because our patients were on ART and subsequently had low to undetectable viral load.

Taken together, our findings identify a novel mechanism of Gal-9 shedding that can contribute to T-cell activation (**S8 Fig**). The interactions between T cells and neutrophils have been only lightly investigated; however, our results suggest a sophisticated interaction between neutrophils and T cells through Gal-9 binding.

With the knowledge that neutrophils can impact T-cell function through the regulated shedding of Gal-9, further investigations are warranted to understand the role of neutrophils in T-cell activation in other acute/chronic conditions. Harnessing the shedding of Gal-9 from neutrophils could be a novel therapeutic target to prevent further HIV replication and enhance CD8^+^ T-cell response.

## Materials and methods

### Study population

Blood samples were taken from 60 healthy subjects (negative for HIV, hepatitis C virus [HCV], and hepatitis B virus [HBV]) and 115 HIV patients (coinfected HIV patients with HCV or HBV were omitted from this study; **S1 Table**). All of the studied patients were on ART. The study was approved by the institutional research review boards at the University of Alberta, and written informed consent was obtained from all the participants in the study with protocol numbers Pro000046064 and Pro000070528. Similar approval with the protocol number AUP00001021 was obtained for the animal studies.

### Cell isolation and culture

Fresh blood samples were collected in EDTA vacutainer blood tubes (BD Biosciences, NJ, USA). PBMCs were isolated by gradient separation using Ficoll-Paque PREMIUM (GE Healthcare, Chicago, USA). The red blood cell pellet containing neutrophils was removed, and red blood cells were lysed using a red blood cell lysis buffer (0.155M NH_4_Cl, 10mM KHCO_3_, and 0.1mM EDTA). Freshly isolated polymorphonuclear (PMN) cells were washed with 4°C PBS and placed in culture media (RPMI-1640 Sigma) supplemented with 10% fetal bovine serum (Sigma, Missouri, USA) and pen/streptomycin (Sigma). Neutrophils were stimulated for 1 to 3 hours at 37°C (5% CO_2_) in vitro by culturing 1 × 10^6^ PMNs in the presence or absence of LPS at100 ng/mL in culture media.

### Flow cytometry

Antibodies used in this study were purchased mainly from BD Biosciences, Thermo Fisher Scientific (MA, USA), and R&D Systems (Minneapolis, USA): human anti-CD3 [SP34-2], anti-CD4 [RPA-T4], anti-CD8 [RPA-T8], anti-CD11b [ICRF44], anti-CD15 [W6D3], anti-CD16 [3G8], anti-CD25 [M-A251], anti-CD32 [FLI8.26], anti-CD38 [HB7], anti-CD44 [515], anti-CD49d [9F10], anti-CD69 [FN50], anti-CD101 [V7.1], anti-CD137 [4B4-1], anti-CD273 [MIH18], anti-CD274 [MIH1], anti-CD366 [7D3], anti-Gal-9 [9M1-3], anti-GLUT1 [FAAB1418F], anti-HLA-DR [G46-6], anti-phospho-LCK(Y505) [4], and anti-VISTA [730804]. Cell viability was measured using an Annexin V Staining Kit (BD Biosciences) and LIVE/DEAD Kit (Life Technologies, CA, USA). ROS was measured using DCFDA / H2DCFDA–Cellular ROS Assay Kit (Abcam, Cambridge, UK). Data were acquired on an LSRFortessa X-20 (BD Biosciences) and analyzed using FlowJo Version 10.5.3.

### Image stream analysis

Stained cells (1 × 10^6^) were fixed in 4% paraformaldehyde, and data were acquired on an Amnis Image Stream Mark II (EMD Millipore, ON, Canada) according to our previous protocols [52,53]. A minimum of 10,000 events were acquired for each sample. Data were analyzed using the IDEAS Analysis Software Package. Neutrophils were identified as having an area >100 and positively stained for CD15. T cells were identified by having an area >80 and stained positively for CD3. Colocalization was measured by calculating the Bright Detail Similarity of the colocalizing probes using the IDEAS wizard. Capping was measured using a delta centroid XY calculation.

### RNA sequencing

RNA libraries were assembled using TruSeq RNA Library Prep Kit v2 (Illumina, San Diego, CA, USA) for sequencing by a NextSeq 500 Instrument (Illumina) as we reported elsewhere [54].

DEGs were identified by subjecting raw counts to analysis using the EdgeR package (3.20.9). Comparisons identified 1,651 genes differentially expressed that had a FDR <0.05 and a log fold change <−2 or >2. Heatmaps were generated using the pHeatmap package. Euclidean distance and Ward’s aggregation criterion. Gene ontologies were generated by inputting a list of DEGs to the gene ontology consortium. Only significant ontologies associated with neutrophil function (FDR <0.05) were considered for analysis. Data generated are publicly available from the Sequence Read Archive (SRA) portal of NCBI under accession number **PRJNA674035.**

The code used for EdgeR DEG RNA-seq analysis and heatmapping can be found in S1 Text. Three healthy study subjects were excluded from the analysis because of the low RNA integrity number (RIN).

### T-cell stimulation studies

Freshly isolated T cells were stimulated with 0.25 μg/mL of anti-CD3 (BD Biosciences) and 0.1 μg/mL of anti-CD28 (BD Biosciences) antibodies according to our previous reports [55,56]. In some studies, recombinant human Gal-9 (GalPharma Co., Ltd, Japan) 1 μg/mL (approximately 31 μM) was added to T cells in the presence/absence of 100 μg/mL of an anti-CD44 blocking antibody (R&D Systems) or anti-CD137/4-1BB (Novus Biologicals). After 48 hours, T-cell activation and phenotype were measured using flow cytometry. In some studies, T-cell stimulation was performed using a trans-well system (Thermo Fisher Scientific) according to the manufacturer’s instructions.

### Glycolysis inhibition

Neutrophil glycolysis was inhibited by the use of several chemical and biological inhibitors. LPS stimulated neutrophils were exposed to 250 μM of the GLUT1 inhibitor phloretin (Sigma). ROS production and Gal-9 shedding were measured using image stream and flow cytometry, respectively. In some studies, ROS production and Gal-9 shedding were quantified in the presence of recombinant IL-10 (R&D Systems).

### CD44 posttranslational modification inhibitors

Neutrophils stimulated for 1 hour with 100 ng/mL of LPS and then were treated in the presence or absence of the DP inhibitors palmityl trifluoromethyl ketone (20 μM) (Cayman Chemical) and methyl arachidonyl fluorophosphonate (5 μM) (Cayman Chemical). To inhibit CaMKII activity, 20 μM of the CaMKII inhibitor CK59 (EMD Millipore) was used. Cells were stained, fixed, and analyzed by flow cytometry and image stream.

### Cytoskeletal inhibitors

Neutrophils were stimulated for 1 hour with 100 ng/mL of LPS before treatment with 100 μM of the RAP-1 inhibitor farnyslthiosialicyclic amide (Millipore Sigma). Ezrin was inhibited by treating neutrophils in the presence of LPS for 1 hour with an ezrin inhibitor NSC668394 (Calbiochem). Upon treatment with the inhibitors, neutrophils were imaged and analyzed by flow cytometry to measure the localization of CD44 and Gal-9.

### Lipid raft depletion

Lipid rafts were depleted by exposing neutrophils to 30 mM MBCD (Sigma) for 20 minutes at 4°C. Neutrophils were analyzed by flow cytometry and imaging to observe the impact of lipid raft depletion on Gal-9 expression and CD44 localization.

### ELISA assay

Plasma was collected from fresh blood samples and frozen at −80°C. Before carrying out the ELISA, the samples were thawed, and the protocol was followed as described by the manufacturer and our report [36]. Gal-9, IL-10, TNFα, IL-4, TGFβ, IL-8, IL-6, IL-7, and IL-13 concentration in the plasma of HIV patients and culture supernatants were measured using Gal-9 and cytokines DuoSet ELISAs (R&D Systems).

### Gene expression analysis

The RNA was isolated from unstimulated, LPS-stimulated neutrophils in the presence or absence of phloretin, CAMKII, Apoc, and IL-10 using the RNAeasy mini kit (Qiagen). cDNA samples for mRNA expression were synthesized using the Quantitec RT kit (Qiagen) according to our previous reports [57,58]. For quantitative real-time PCR, samples were run in duplicate using Quantitect LGALS9 primers (QT02309545), and Beta-2-microglobulin (QT01149547) was used as a reference gene (Qiagen). Samples were analyzed by the CFX96 Touch Real-Time PCR Detection System (Bio-Rad). Data analysis was carried out using the 2^−ddCT^ method.

### Statistical analysis

Statistical analysis was performed by utilizing Prism version 8 using appropriate statistical tests depending on the data. Differences between groups were evaluated using 1-way ANOVA followed by Tukey test for multiple comparisons. Results are expressed as mean± SEM. *P* value <0.05 was considered as statistically significant.

Additionally, as previously mentioned, all RNA-seq statistical analysis was performed using the package edgeR. All statistically significant values were identified as having a *P* value FDR of <0.05.

## Supporting information

S1 Fig **(A)** Volcano plot showing the difference between DEGs up-regulated in neutrophils from HCs compared to HIV patients with low CD4 count. **(B)** Volcano plot showing the difference between DEGs up-regulated in neutrophils from HCs compared to HIV patients with high CD4 count. **(C)** Volcano plot showing the difference between DEGs up-regulated in neutrophils of HIV patients with low CD4 count compared to high CD4 count. The underlying data can be found from the SRA portal of NCBI under accession number **PRJNA674035**. DEG, differentially expressed gene; HC, healthy control; SRA, Sequence Read Archive.
(TIF)

S2 Fig **(A)** Data representing gene ontologies associated with clusters 2 and 4 in Fig 1. **(B)** Heatmap representing gene expression associated with the gene ontology of neutrophil activation. The underlying data can be found from the SRA portal of NCBI under accession number **PRJNA674035**. **(C)** Experimental scheme for data represented in Fig 2. SRA, Sequence Read Archive.
(TIF)

S3 Fig **(A)** Representative plots and **(B)** cumulative data showing the percent CD4^+^ T cells co-expressing CD25 and HLA-DR in the presence or absence of neutrophils from HIV patients with low CD4 T-cell count. **(C)** Representative plots and **(D)** cumulative data showing the percent of CD8^+^ T cells co-expressing CD25 and HLA-DR in the presence or absence of neutrophils from HIV patients with low CD4 T-cell count. **(E)** Representative plots and (**F**) cumulative data showing the percent CD4^+^ T cells co-expressing HLA-DR and CD38 in the presence or absence of neutrophils from HIV patients with low CD4 T-cell count. **(G)** Representative plots and **(H)** cumulative data of CD8^+^ T cells co-expressing HLA-DR and CD38 in the presence or absence of neutrophils from HIV patients with low CD4 T-cell count. The underlying data can be found in S2 Data.
(TIF)

S4 Fig **(A)** Heatmaps showing the expression of T-cell modulating cytokine transcripts in HCs, HIV patients with high or low CD4 T-cell count. **(B)** Heatmaps indicating the magnitude of co-inhibitory and co-stimulatory receptors expression in neutrophils of HCs, HIV patients with high and low CD4 T-cell count. The underlying data can be found from the SRA portal of NCBI under accession number **PRJNA674035**. **(C)** Representative plots showing the gating strategy for neutrophils identification and co-inhibitory receptors expression (e.g., Gal-9) on neutrophils from an HC and an HIV-infected individual with low CD4 T-cell count. Gal-9, Galectin-9; HC, healthy control; SRA, Sequence Read Archive.
(TIF)

S5 Fig **(A)** Representative plots of HLA-DR expression and **(B)** cumulative data in HCs and HIV patients. **(C)** Representative plots of PDL-1/PDL-2 expression and **(D, E)** cumulative data in HCs and HIV patients. **(F)** Representative plots of VISTA expression and **(G)** cumulative data in HCs and HIV patients. **(H)** Cumulative data of CD40 and **(I)** TIM-3 expression in neutrophils from HCs and HIV patients. **(J)** Representative plots and **(K)** cumulative data of CD69/CD38 expression in CD4^+^ T cells from HIV-infected individuals with low CD4 count using the trans-well system in the presence/absence of autologous neutrophils and or lactose. **(L)** Representative plots and **(M)** cumulative data of CD69/CD38 expression in CD8^+^ T cells from HIV-infected individuals with low CD4 count using the trans-well system in the presence/absence of neutrophils and or lactose. The underlying data can be found in S2 Data. HC, healthy control.
(TIF)

S6 Fig **(A)** Image stream gating strategy for neutrophil colocalization of Gal-9 and CD44. **(B)** Representative plots of Gal-9 expression on neutrophils once unstimulated (Unstim.) or stimulated with LPS (+LPS). **(C)** Relative expression of DDIT4 in neutrophils from HCs and HIV patients with low and high CD4 T-cell count. **(D)** Expression of Gal-9 in murine neutrophils. **(E)** Cumulative results of murine neutrophil Gal-9 expression. **(F)** Image of murine neutrophil expression of Gal-9 and CD44. BF. The underlying data can be found in S2 Data. BF, Bright Field; Gal-9, Galectin-9; HC, healthy control; LPS, lipopolysaccharide.
(TIF)

S7 Fig Gene expression in neutrophils associated with the gene ontologies, **(A)** protein DP, and **(B)** glycolytic process. The underlying data can be found from the SRA portal of NCBI under accession number **PRJNA674035**. DP, depalmitoylation; SRA, Sequence Read Archive.
(TIF)

S8 Fig Visual representation of the mechanism of Gal-9 shedding from neutrophils. Gal-9, Galectin-9.
(TIF)

S1 Table Participants’ demographic and clinical data. (PDF)

S1 Text Supporting information methods used for RNA-seq. RNA-seq, RNA sequencing. (PDF)

S1 Data Underlying data for Figs 2–8. (XLSX)

S2 Data Underlying data for S3, S5 and S6 Figs. (XLSX)

## Abbreviations

APC: antigen-presenting cell
Apoc: Apocynin
ART: antiretroviral therapy
CaMKII: calcium/calmodulin-dependent protein kinase II
DEG: differentially expressed gene
DP: depalmitoylation
ERK: extracellular signal–regulated kinase
FcγR: Fc gamma receptor
FDR: false discovery rate
Gal-9: Galectin-9
GLUT1: glucose transporter 1
HBV: hepatitis B virus
HC: healthy control
HCV: hepatitis C virus
IFNγ: interferon gamma
IL: interleukin
LDW: low density weight
LPS: lipopolysaccharide
MAPK: mitogen-activated protein kinase
MBCD: methyl-beta-cyclodextran
NFAT: nuclear factor of activated T cells
NK: natural killer
PBMC: peripheral blood mononuclear cell
PCA: principal component analysis
PDI: protein disulfide isomerase
PKC: protein kinase C
PMN: polymorphonuclear
PPP: pentose–pyruvate phosphatase pathway
RIN: RNA integrity number
RNA-seq: RNA sequencing
ROS: reactive oxygen species
SARS-CoV-2: Severe Acute Respiratory Syndrome Coronavirus 2
SRA: Sequence Read Archive
TGFβ: transforming growth factor beta
TNFα: tumor necrosis factor alpha
Treg: regulatory T cell
