# Supplementary figures and images for: Neutrophils promote T-cell activation through the regulated release of CD44-bound Galectin-9 from the cell surface during HIV infection
Source: PLoS Biol. 2021 Aug 19;19(8):e3001387. doi: 10.1371/journal.pbio.3001387 (PMC8407585; doi:10.1371/journal.pbio.3001387)

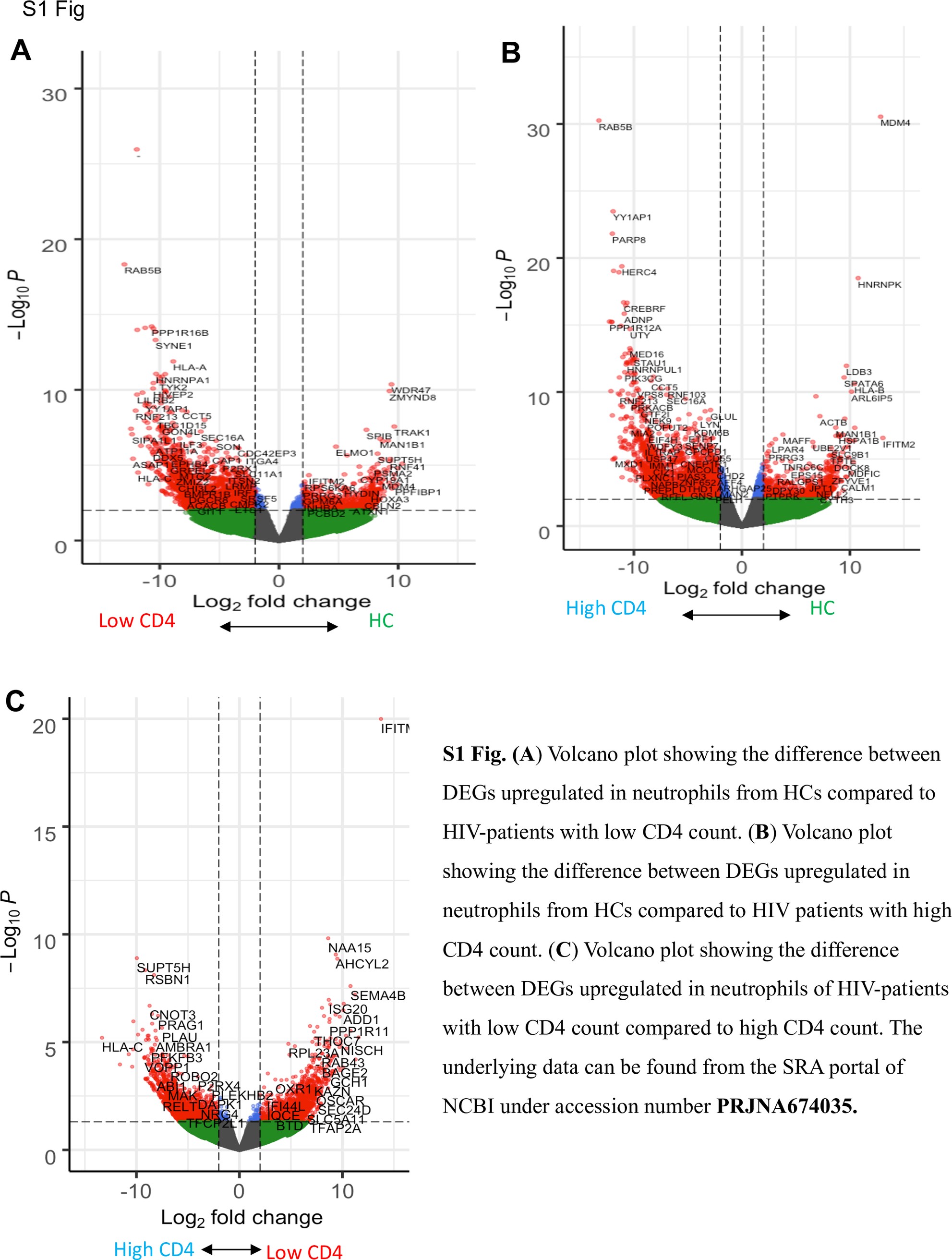

Supplement: S1 Fig — (A) Volcano plot showing the difference between DEGs up-regulated in neutrophils from HCs compared to HIV patients with low CD4 count. (B) Volcano plot showing the difference between DEGs up-regulated in neutrophils from HCs compared to HIV patients with high CD4 count. (C) Volcano plot showing the difference between DEGs up-regulated in neutrophils of HIV patients with low CD4 count compared to high CD4 count. The underlying data can be found from the SRA portal of NCBI under accession number PRJNA674035. DEG, differentially expressed gene; HC, healthy control; SRA, Sequence Read Archive. (TIF) [file pbio.3001387.s001.tif]

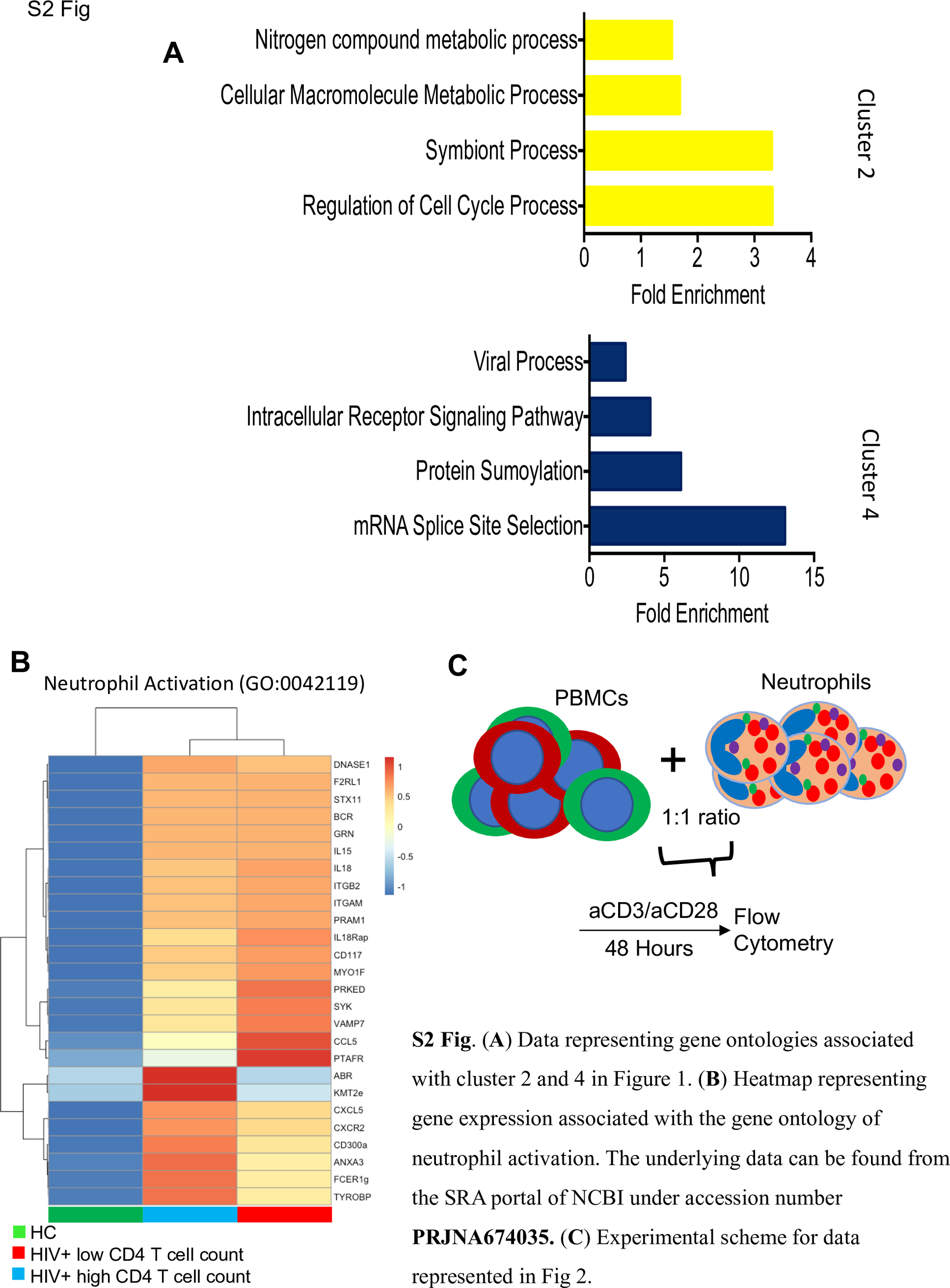

Supplement: S2 Fig — (A) Data representing gene ontologies associated with clusters 2 and 4 in Fig 1. (B) Heatmap representing gene expression associated with the gene ontology of neutrophil activation. The underlying data can be found from the SRA portal of NCBI under accession number PRJNA674035. (C) Experimental scheme for data represented in Fig 2. SRA, Sequence Read Archive. (TIF) [file pbio.3001387.s002.tif]

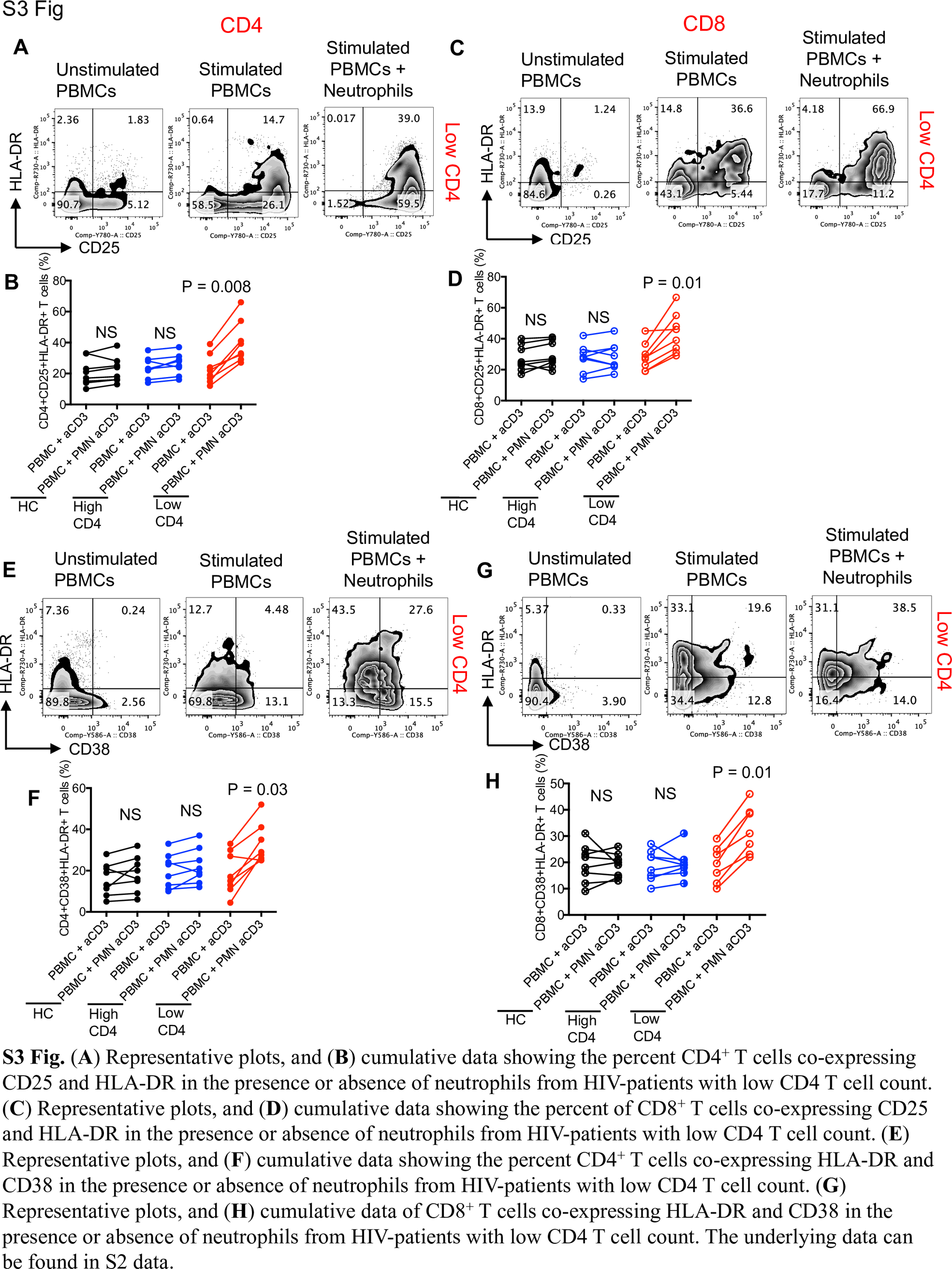

Supplement: S3 Fig — (A) Representative plots and (B) cumulative data showing the percent CD4+ T cells co-expressing CD25 and HLA-DR in the presence or absence of neutrophils from HIV patients with low CD4 T-cell count. (C) Representative plots and (D) cumulative data showing the percent of CD8+ T cells co-expressing CD25 and HLA-DR in the presence or absence of neutrophils from HIV patients with low CD4 T-cell count. (E) Representative plots and (F) cumulative data showing the percent CD4+ T cells co-expressing HLA-DR and CD38 in the presence or absence of neutrophils from HIV patients with low CD4 T-cell count. (G) Representative plots and (H) cumulative data of CD8+ T cells co-expressing HLA-DR and CD38 in the presence or absence of neutrophils from HIV patients with low CD4 T-cell count. The underlying data can be found in S2 Data. (TIF) [file pbio.3001387.s003.tif]

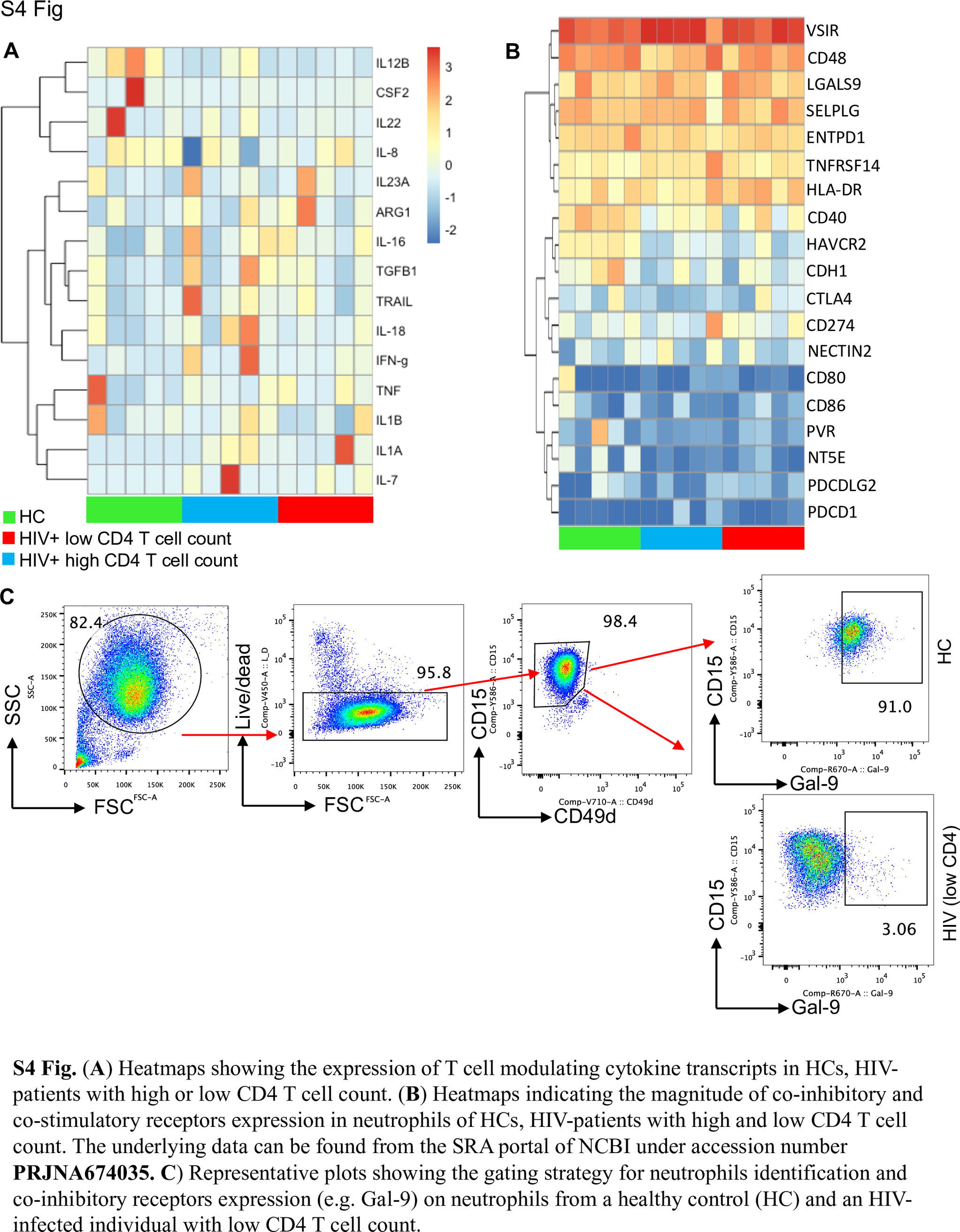

Supplement: S4 Fig — (A) Heatmaps showing the expression of T-cell modulating cytokine transcripts in HCs, HIV patients with high or low CD4 T-cell count. (B) Heatmaps indicating the magnitude of co-inhibitory and co-stimulatory receptors expression in neutrophils of HCs, HIV patients with high and low CD4 T-cell count. The underlying data can be found from the SRA portal of NCBI under accession number PRJNA674035. (C) Representative plots showing the gating strategy for neutrophils identification and co-inhibitory receptors expression (e.g., Gal-9) on neutrophils from an HC and an HIV-infected individual with low CD4 T-cell count. Gal-9, Galectin-9; HC, healthy control; SRA, Sequence Read Archive. (TIF) [file pbio.3001387.s004.tif]

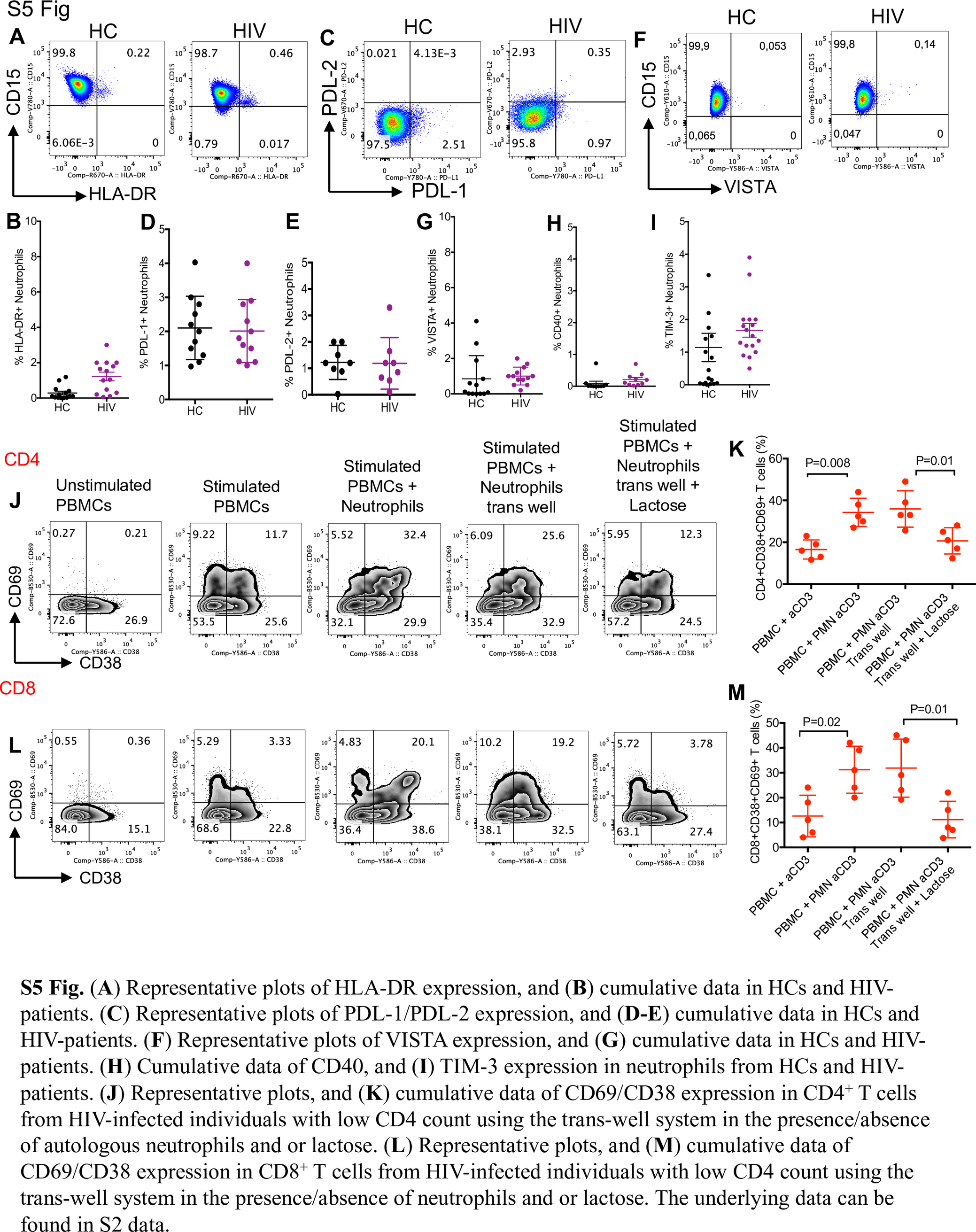

Supplement: S5 Fig — (A) Representative plots of HLA-DR expression and (B) cumulative data in HCs and HIV patients. (C) Representative plots of PDL-1/PDL-2 expression and (D, E) cumulative data in HCs and HIV patients. (F) Representative plots of VISTA expression and (G) cumulative data in HCs and HIV patients. (H) Cumulative data of CD40 and (I) TIM-3 expression in neutrophils from HCs and HIV patients. (J) Representative plots and (K) cumulative data of CD69/CD38 expression in CD4+ T cells from HIV-infected individuals with low CD4 count using the trans-well system in the presence/absence of autologous neutrophils and or lactose. (L) Representative plots and (M) cumulative data of CD69/CD38 expression in CD8+ T cells from HIV-infected individuals with low CD4 count using the trans-well system in the presence/absence of neutrophils and or lactose. The underlying data can be found in S2 Data. HC, healthy control. (TIF) [file pbio.3001387.s005.tif]

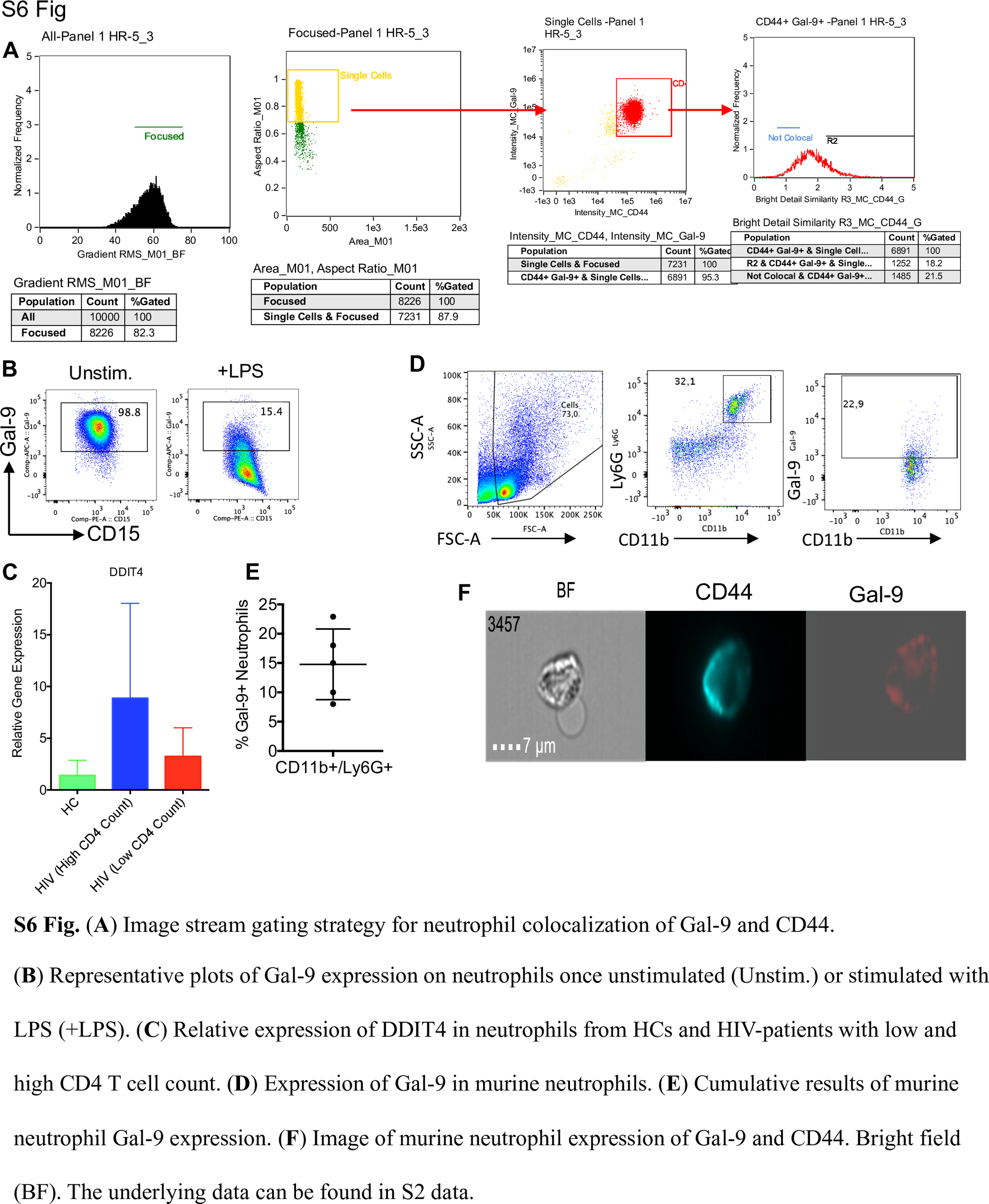

Supplement: S6 Fig — (A) Image stream gating strategy for neutrophil colocalization of Gal-9 and CD44. (B) Representative plots of Gal-9 expression on neutrophils once unstimulated (Unstim.) or stimulated with LPS (+LPS). (C) Relative expression of DDIT4 in neutrophils from HCs and HIV patients with low and high CD4 T-cell count. (D) Expression of Gal-9 in murine neutrophils. (E) Cumulative results of murine neutrophil Gal-9 expression. (F) Image of murine neutrophil expression of Gal-9 and CD44. BF. The underlying data can be found in S2 Data. BF, Bright Field; Gal-9, Galectin-9; HC, healthy control; LPS, lipopolysaccharide. (TIF) [file pbio.3001387.s006.tif]

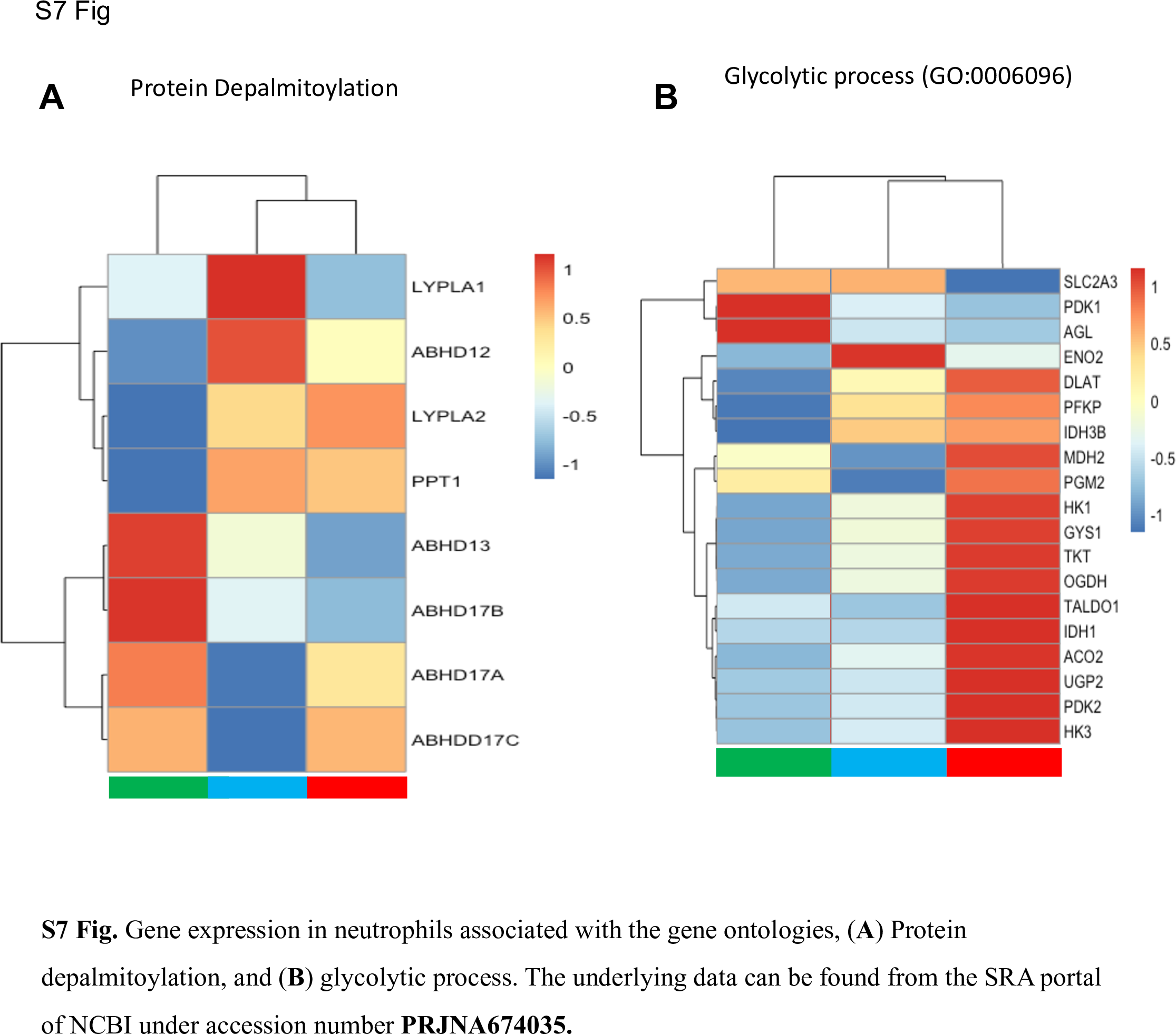

Supplement: S7 Fig — Gene expression in neutrophils associated with the gene ontologies, (A) protein DP, and (B) glycolytic process. The underlying data can be found from the SRA portal of NCBI under accession number PRJNA674035. DP, depalmitoylation; SRA, Sequence Read Archive. (TIF) [file pbio.3001387.s007.tif]

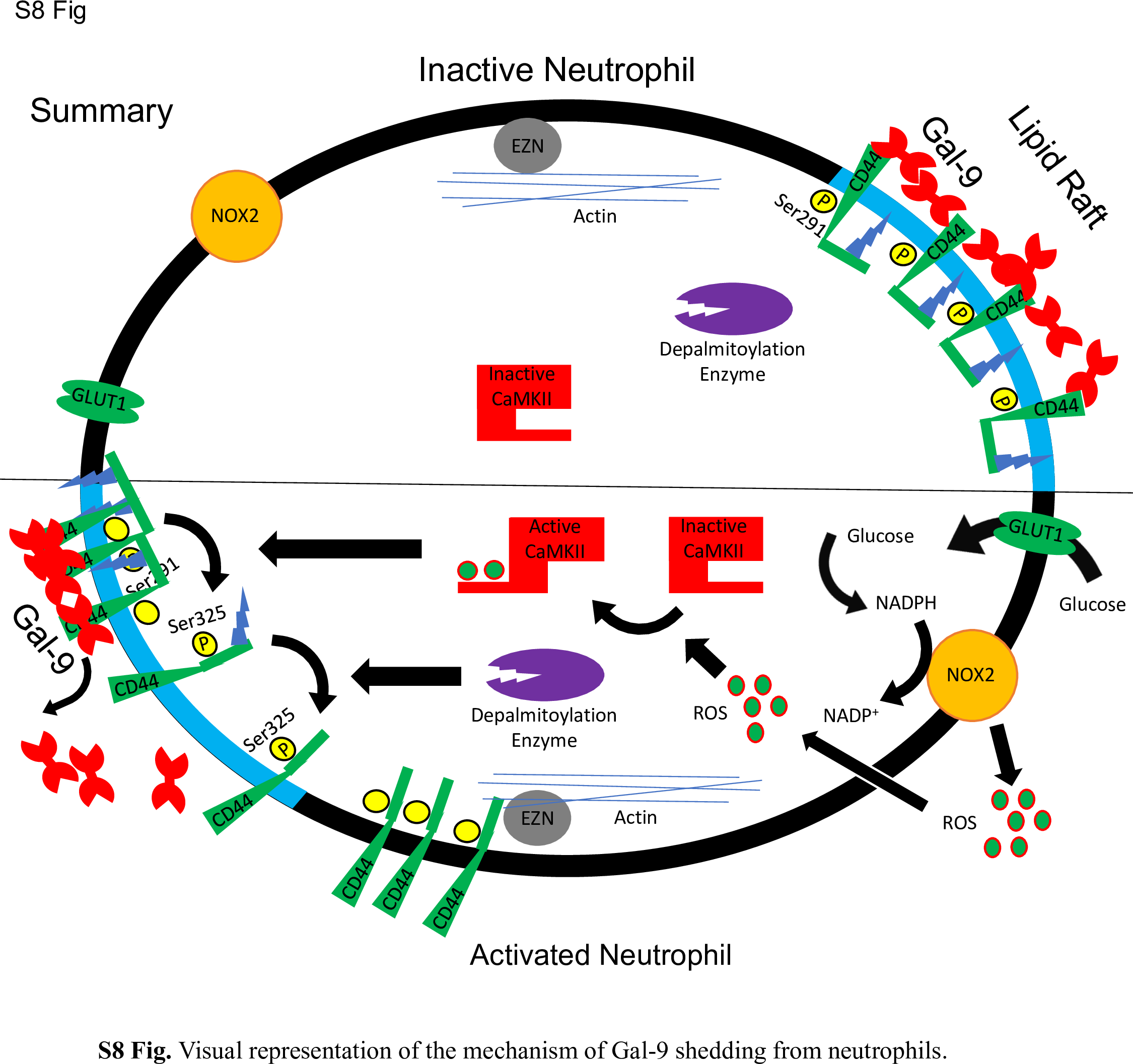

Supplement: S8 Fig — Gal-9, Galectin-9. (TIF) [file pbio.3001387.s008.tif]
