# Supplementary material for: Neutrophils promote T-cell activation through the regulated release of CD44-bound Galectin-9 from the cell surface during HIV infection
Source: PLoS Biol. 2021 Aug 19;19(8):e3001387. doi: 10.1371/journal.pbio.3001387 (PMC8407585; doi:10.1371/journal.pbio.3001387)
Supplement: S1 Table — (PDF) [file pbio.3001387.s009.pdf]

Supplementary Table 1 Participants demographic and clinical data.

**PTID**	**Sex**	**Plasma viral load (copies/mL-^{1})**	**CD4 count (mL^{-1})**	**On ART**
ART-1	M	<30	720	yes
ART-2	M	<30	570	yes
ART-3	M	<30	1080	yes
ART-4	F	<30	350	yes
ART-5	M	1399	800	yes
ART-6	M	<30	720	yes
ART-7	M	<30	830	yes
ART-8	M	<30	460	yes
ART-9	M	<30	470	yes
ART-10	F	<30	310	yes
ART-11	M	<30	350	yes
ART-12	M	<30	850	yes
ART-13	M	<30	740	yes
ART-14	M	<30	680	yes
ART-15	M	<30	470	yes
ART-16	M	<30	550	yes
ART-17	F	208	389	yes
ART-18	M	<30	320	yes
ART-19	M	<30	580	yes
ART-20	F	<30	590	yes
ART-21	M	<30	680	yes
ART-22	M	<30	370	yes
ART-23	M	<30	420	yes
ART-24	M	<30	210	yes
ART-25	M	<30	500	yes
ART-26	M	<30	640	yes
ART-27	M	<30	260	yes
ART-28	F	<30	700	yes
ART-29	F	<30	720	yes
ART-30	M	<30	910	yes
ART-31	F	168	290	yes
ART-32	F	<30	180	yes
ART-33	M	<30	680	yes
ART-34	M	<30	510	yes
ART-35	M	<30	1210	yes
ART-36	F	<30	550	yes
ART-37	F	60	910	yes
ART-38	F	<30	190	yes
ART-39	M	<30	340	yes
ART-40	F	160	60	yes
ART-41	M	<30	390	yes

ART-42	M	<30	250	yes
ART-43	M	<30	520	yes
ART-44	M	<30	610	yes
ART-45	M	<30	360	yes
ART-46	M	<30	290	yes
ART-47	M	<30	250	yes
ART-48	M	<30	260	yes
AET-49	M	<30	342	yes
ART-50	F	<30	457	yes
ART-51	M	<30	632	yes
ART-52	M	540	578	yes
ART-53	F	990	350	yes
ART-54	M	<30	602	yes
ART-55	M	<30	575	yes
ART-56	M	800	251	yes
ART-57	M	<30	790	yes
ART-58	M	<30	850	yes
ART-59	M	125	783	yes
ART-60	M	<30	765	yes
ART-61	M	<30	562	yes
ART-62	F	<30	690	yes
ART-63	F	<30	720	yes
ART-64	F	89	307	yes
ART-65	F	<30	333	yes
ART-66	M	403	463	yes
ART-67	F	<30	305	yes
ART-68	M	<30	447	yes
ART-69	M	<30	479	yes
ART-70	M	<30	798	yes
ART-71	M	733	156	yes
ART-72	M	172	382	yes
ART-73	F	<30	390	yes
ART-74	M	<30	335	yes
ART-74	M	<30	414	yes
ART-76	M	<30	259	yes
ART-77	F	<30	551	yes
ART-78	M	153	329	yes
ART-79	M	<30	239	yes
ART-80	M	<30	396	yes
ART-81	M	<30	321	yes
ART-82	M	<30	408	yes
ART-83	F	<30	637	yes
ART-84	M	<30	748	yes
ART-85	M	476	556	yes

ART-86	M	<30	621	yes
ART-87	M	<30	547	yes
ART-88	M	167	448	yes
ART-89	F	<30	787	yes
ART-90	M	<30	357	yes
ART-91	F	<30	312	yes
ART-92	M	<30	741	yes
ART-93	M	<30	458	yes
ART-94	M	<30	620	yes
ART-95	F	<30	280	yes
ART-96	M	<30	254	yes
ART-97	M	<30	572	yes
ART-98	M	650	480	yes
ART-99	M	<30	658	yes
ART-100	M	<30	312	yes
ART-101	F	1234	274	yes
ART-102	M	<30	783	yes
ART-103	M	<30	765	yes
ART-104	M	200	262	yes
ART-105	F	<30	490	yes
ART-106	F	<30	710	yes
ART-107	M	<30	317	yes
ART-108	M	<30	233	yes
ART-109	F	250	473	yes
ART-110	M	<30	325	yes
ART-111	F	<30	520	yes
ART-112	F	<30	650	yes
ART-113	M	<30	190	yes
ART-114	M	<30	365	yes
ART-115	F	<30	325	yes

On antiretroviral therapy (ART)
Male (M) and female (F)
Healthy controls (n=60) were 33 males and 27 females.
