# Supplementary material for: Neutrophils promote T-cell activation through the regulated release of CD44-bound Galectin-9 from the cell surface during HIV infection
Source: PLoS Biol. 2021 Aug 19;19(8):e3001387. doi: 10.1371/journal.pbio.3001387 (PMC8407585; doi:10.1371/journal.pbio.3001387)
Supplement: S1 Text — (PDF) [file pbio.3001387.s010.pdf]

Neutrophils promote T-cell activation through the regulated release of CD44-bound Galectin-9 from the cell surface during HIV-infection

Dunsmore et al.

Supplemental text 1: The code used for EdgeR DEG RNA sequencing analysis and heatmapping.

```
# Package Load

library(pheatmap)

library(edgeR)

library(cluster)

library(statmod)

# Set Directory

setwd("~/Documents/RNA Sequencing/Neutrophil Phenotyping/")

# Read Tab Deliminanted file of RAW COUNTS - THIS DOES NOT MEAN
TPM/CPM/RPKM/htsqct/fastqc

Rawdata <- read.delim("HC CD4 Low and CD4 High.txt", row.names = "target_id")

# Group Samples based on Subject

length(Rawdata)

# DGEList Conversion

y <- DGEList(counts=Rawdata[,])

# Filtering and Normalizing

y$samples

# Normalize the data by "Relative Log Expression" (RLE = Log2)

y <- calcNormFactors(y,method=c("RLE"))

#How many surviving samples

head(y$samples)

# Data Exploration
```

```
# Multidimensional Scaling

keep <- rowSums(cpm(y)>2) >= 5

y <- y[keep, , keep.lib.sizes=FALSE]

plotMDS(y, top = 5000, col=c(rep("black",5), rep("red",5), rep("blue",5)), gene.selection =
"common", pch = 16, cex = 2)

#Conditioning - Expreimental treatments to samples are taken into account for differential
equation analysis

condition <- factor(c("HC", "HC", "HC", "HC", "HC",
                      "CD4 High", "CD4 High", "CD4 High", "CD4 High", "CD4 High",
                      "CD4 Low", "CD4 Low", "CD4 Low", "CD4 Low", "CD4 Low"))

# Estimate Dispersion

# Organize the data by subject (individual/patient/mouse/etc.)

subject <- factor(c(1,2,3,4,5,6,7,8,9,10,11,12,13,14,15))

# Ensure the appropriate subject and conditions match the sample

data.frame(group=colnames(y),subject,condition)

# Make the expreimental design that will be utilized by the dispersion analysis

design <- model.matrix(~condition)

# Subject data to GLM Dispersion - Utilizes the

# Cox-Reid profile adjusted likelihood method (CR) to perform generalized linear model with

# design previously mentioned

# Subject data to GLM Trended Dispersion

countTable <- estimateGLMTrendedDisp(y, design)

names (countTable)
```

```
# Subject data to GLM Tagwise Dispersion

summary(countTable$trended.dispersion)

countTable <- estimateGLMTagwiseDisp(countTable, design)

names (countTable)

summary( countTable$tagwise.dispersion )

# Subject Data to GLM Common Dispersion - Verbose

countTable <- estimateGLMCommonDisp(countTable, design, verbose=TRUE)

names (countTable)

summary( countTable$common.dispersion )

# Plot the Biological Coefficient of Variation

plotBCV(countTable)

# Plot Mean Variability to demonstrate the variation

meanVarPlot <- plotMeanVar( countTable ,show.raw.vars=TRUE , show.tagwise.vars=TRUE ,

                    show.binned.common.disp.vars=FALSE , show.ave.raw.vars=FALSE ,

                    NBline = TRUE , nbins = 100 ,

                    #these are arguments about what is plotted

                    pch = 16 , xlab ="Mean Expression (Log10 Scale)" ,

                    ylab = "Variance (Log10 Scale)" , main = "Mean-Variance Plot" )

# Differential Expression

# Fit the GLM data to the analysis

fit <- glmFit(countTable, design, robust=TRUE)

# Perform likelyhood ratio test on the GLM fit data -
```

```
# This test reads out Log Fold Change, Log CPM, Likelyhood Ratio, P value, and False
Discovery Rate

LRT <- glmLRT(fit, coef =2:3)

topTags(LRT,n=10)

# Export Stats findings to a table (n = number of genes being tested (Rows in Rawdata))

Take <- topTags(LRT,n=200000, sort.by = "none")

write.table(Take, 'Stats Neutrophils High v Low CD4 Coef2:3.txt', sep="\t")

top <- rownames(topTags(LRT))

cpm(y)[top,]

summary(decideTests(LRT))

# Plot Mean-Difference

plotMD(LRT)

# Add a line at logFC -2 and +2 as these are the cutoffs for heatmapping later on.

abline(h=c(-2,2), v=(107), col="green")

# Perform FDR analysis using the Beyer-Hardwick Method

FDR <- p.adjust(LRT$table$PValue, method="BH")

# FDR cutoff to demonstrate significant data

FDRajust <- sum(FDR < 0.05)

# How many genes are significantly different

summary(decideTests(FDR))

# Session info - keep a log of this with publishable formal analysis

sessionInfo()

#Heatmapping
```

```
# Package Load

library(pheatmap)

library(edgeR)

library(cluster)

library(statmod)

library(factoextra)

library(FactoMineR)

library(ggpubr)

# Set Directory

setwd("~/Documents/RNA Sequencing/Neutrophil Phenotyping/")

# Read Tab Deliminanted file of RAW COUNTS - THIS DOES NOT MEAN

TPM/CPM/RPKM/htsqct/fastqc

Rawdata <- read.delim("DupGone.txt", row.names = "target_id")

PCARaw <- prcomp(Rawdata)

fviz_pca_ind(Rawdata)

# DGEList Conversion

y <- DGEList(counts=Rawdata[,])

# Filtering and Normalizing

y$samples

keep <- rowSums(cpm(y)>2) >= 5

y <- y[keep, , keep.lib.sizes=TRUE]

keep <- rowSums(cpm(y)>2) <= 10

y <- y[keep, , keep.lib.sizes=FALSE]
```

```
# Heat Mapping DGE Data

logcpm <- cpm(y, prior.count = 0, log=FALSE)



cal_z_score <- function(x){

  (x - mean(x)) / sd(x)

}

data_subset_norm <- t(apply(logcpm, 1, cal_z_score))

res <- pheatmap(data_subset_norm, show_rownames = F, clustering_method = "ward.D2",

clustering_distance_rows = "euclidean", clustering_distance_cols = "euclidean")

scaled <- scale(logcpm)

res <- pheatmap(logcpm, show_rownames = F, cutree_cols = 1, clustering_method = "ward.D2",

clustering_distance_rows = "euclidean", clustering_distance_cols = "euclidean")

logcpmClust <- cbind(data_subset_norm,

                     cluster = cutree(res$tree_row, k = 5))

pheatmap(logcpmClust, cutree_cols = 4, show_rownames = F,

        clustering_method = "ward.D2", clustering_distance_rows = "euclidean",

clustering_distance_cols = "euclidean")

write.table(logcpmClust, ‘2.txt', sep="\t")
```
